# Fecal Microbiota Transplantation from Young-Trained Donors Improves Cognitive Function in Old Mice Through Modulation of the Gut-Brain Axis

**DOI:** 10.14336/AD.2024.1089

**Published:** 2025-12-26

**Authors:** Camila Cerna, Nicole Vidal-Herrera, Francisco Silva-Olivares, Daniela Álvarez, Camila González-Arancibia, Miltha Hidalgo, Pabla Aguirre, José González-Urra, Camila Astudillo-Guerrero, Michel Jara, Omar Porras, Gonzalo Cruz, Christian Hodar, Paola Llanos, Pamela Urrutia, Claudia Ibacache-Quiroga, Yulia Nevzorova, Francisco Javier Cubero, Marco Fuenzalida, Samanta Thomas-Valdés, Gonzalo Jorquera

**Affiliations:** ^1^Center for Neurobiology and Integrative Physiopathology (CENFI), Science Faculty, Universidad de Valparaíso, Valparaíso, Chile.; ^2^Institute of Nutrition and Food Technology (INTA), Universidad de Chile, Santiago, Chile.; ^3^Physiology Institute, Science Faculty, Universidad de Valparaíso, Valparaíso, Chile.; ^4^Institute for Research in Dental Sciences, Faculty of Dentistry, Universidad de Chile, Santiago, Chile.; ^5^Geroscience Center for Brain Health and Metabolism, Santiago, Chile.; ^6^Centro de MicroBioinnovación (CMBi), School of Nutrition and Dietetics, Faculty of Pharmacy, Universidad de Valparaíso, Valparaíso, Chile.; ^7^Department of Immunology, Ophthalmology and ENT, Complutense University School of Medicine Madrid, Spain.; ^8^Instituto de Investigación Sanitaria Gregorio Marañón (IiSGM), Madrid, Spain.; ^9^Centre for Biomedical Research, Network on Liver and Digestive Diseases (CIBEREHD), Madrid, Spain.; ^10^ Millennium Nucleus of Neuroepigenetics and Plasticity (EpiNeuro), Santiago, Chile.

**Keywords:** Gut microbiota, aging, exercise, synaptic plasticity, gut permeability, Akkermansia

## Abstract

The gut-brain axis is a bidirectional communication pathway that modulates cognitive function. A dysfunctional gut-brain axis has been associated with cognitive impairments during aging. Therefore, we propose evaluating whether modulation of the gut microbiota through fecal microbiota transplantation (FMT) from young-trained donors (YT) to middle-aged or aged mice could enhance brain function and cognition in old age. Twelve-month-old male mice received an initial FMT from YT (YT-Tr) or age-matched donors (Auto-Tr) following antibiotic treatment. Three months later, the mice received a second FMT as reinforcement. Additionally, 18-month-old mice received Auto-Tr, YT-Tr, or FMT from young sedentary donors (YS-Tr). Cognitive function was assessed using novel object recognition and object location memory tests. Long-term potentiation (LTP) in hippocampal brain slices was studied, while neuroinflammation and synaptic plasticity were analyzed in hippocampal samples via qPCR and immunoblot. Gut permeability was evaluated in ileum and colon sections, serum samples were analyzed for cytokine levels, and fecal samples were used to measure short-chain fatty acid (SCFA) levels and perform 16S rRNA gene sequencing. We observed that YT-Tr, whether performed in middle age or old age, improved cognitive function in aged mice. Recognition and spatial memory were significantly enhanced in YT-Tr mice compared to Auto-Tr and YS-Tr groups. Intact LTP was observed in YT-Tr mice at 18 months of age, whereas LTP was impaired in the Auto-Tr group. Neuroinflammation was reduced, and synaptic plasticity modulators such as PSD-95 and FNDC5/Irisin were upregulated in the hippocampus of YT-Tr mice compared to both YS-Tr and Auto-Tr groups. A significant reduction in ileal and colon permeability was detected in YT-Tr animals, along with elevated cecal levels of butyrate and valerate compared to Auto-Tr. Moreover, YT-Tr decreased pro-inflammatory factors and increased anti-inflammatory factors in the serum of aged mice. Beta diversity analysis revealed significant differences in microbial community composition between YT-Tr and Auto-Tr animals, with higher abundances of *Akkermansia*, *Prevotellaceae_ UCG-001*, and *Odoribacter* in YT-Tr mice. In conclusion, our study demonstrates that FMT from young-trained donors improves cognitive function and synaptic plasticity by modulating gut permeability, inflammation, SCFA levels, and gut microbiota composition in aged mice.

## INTRODUCTION

Aging is a natural time-dependent process characterized by the body’s limited capacity to regenerate itself. This life stage commonly exhibits many physiological changes including metabolic, cognitive, and motor functions declines [1]. Additionally, elderly subjects show a reduction in gut microbiota diversity and a variation in the relative abundance of certain species, known as gut dysbiosis [2]. This condition leads to poor intestinal barrier selectivity to pathogens, resulting in low-grade chronic inflammation and dysregulation of the immune system [2, 3]. Gut dysbiosis, inflammation and immune dysregulation have been identified in almost all chronic and neurodegenerative diseases, including aging-associated cognitive decline [4]. Aged gut microbial ecosystems exhibit lower proportion of Firmicutes/Bacteroidetes ratio, increment in inter-individual variability, higher relative abundance of Proteobacteria, lower content of beneficial taxa such as *Bifidobacterium*, higher susceptibility to pathogen infection, and weakened gut mucosal barrier [2-4]. All these changes are exacerbated by long-term antibiotics treatment, and low-quality diet with inadequate fiber, micronutrients, and phytochemicals consumption [5]. Elderly subjects are often on medication and have dietary restrictions that reduce colonic fermentation capacity, leading to lower levels of microbiota-derived metabolites [2, 3]. Among these are short-chain fat acids (SCFA), bacterial metabolites that benefit different organs, including the brain and muscles [5].

The gut-brain axis is a bidirectional pathway, where microbiota diversity and its derived metabolites play a central role in promoting brain health. Therefore, disturbances in this crosstalk have been associated with brain diseases [5]. Indeed, a decrease in SCFA levels in both blood and feces has been reported during aging [6]. On the contrary, higher levels of acetate, propionate, butyrate, and valerate have been associated with cognitive improvements in animal models and humans[2]. Increased *Streptococcus* and *Acinetobacter*, and decreased *Bifidobacterium* and *Lachnospira* have been reported in the gut microbiota of individuals with dementia [7], while some SCFA-producing bacteria like *Roseburia* and *Faecalibacterium prausnitzii* are underrepresented [8]. These observations imply a link between reduced SCFAs, alterations in bacterial composition, and the onset of brain degenerative diseases.

Recent studies suggest that physical exercise significantly impacts the microbiome, resulting in increased levels of specific bacteria associated with cognitive well-being. Endurance-based training in lean volunteers leads to an increase in *Faecalibacterium* and *Lachnospira*, and a subsequent rise in fecal SCFAs, along with a decrease in *Bacteroides* genus [9]. Conversely, researchers observed a decrease in *Faecalibacterium* and an increase in *Bacteroides* and *Collinsella* populations in sedentary obese volunteers [9]. These microbial changes associated to exercise are believed to contribute to the production of neuroprotective SCFA, antioxidant enzymes, and anti-inflammatory cytokines, thereby providing crucial support for cognitive health. Gut microbiota modulation through exercise may be an alternative to maintaining brain health during aging. However, chronic age-related diseases can limit the ability to engage in regular physical activity, significantly impacting mobility and physical function [10], and limiting the ability to participate in regular exercise routines. On the other hand, early adulthood has been associated with a high diversity of microorganisms in the gut and an increased representation of bacteria associated with good health in the host [2]. In young adults, the intestinal lumen and mucin layer, both harbor a varied community of commensal microbes, coexisting symbiotically with the host [11]. In contrast, the dysbiosis observed in older adults is characterized by an accumulation of potentially pro-inflammatory commensals and a reduction in the relative abundance of beneficial microbes [12].

Fecal microbiota transplantation (FMT) is used to reshape the recipient’s gut microbiota, to modify its composition and achieve a therapeutic effect. FMT is employed to treat refractory *Clostridium difficile* infection [13], but new uses have been proposed [13]. Here, we investigate if modulation of the gut microbiota through FMT, utilizing middle-aged or aged mice as recipients and young-trained donors as sources, could enhance brain function and cognition during aging. Our selection of donors is based on the positive characteristics of the host’s health that have been identified in the gut microbiota of healthy and trained young adults. This research seeks to enhance our understanding of the complex relationship between gut microbiota, aging, and cognitive health.

## MATERIALS AND METHODS

### Animals

C57BL/6J male mice were obtained from the Animal Facility of the Faculty of Sciences at the Universidad de Valparaíso or from Animal Facility of the Chilean Public Health Institute (ISP). Animals were housed under controlled parameters (room temperature was maintained at 23°C, with a 12:12 h light-dark cycle). The animals were fed a normal control diet (NCD), which consisted of 10% of dietary energy from fat, 20% from proteins, and 70% from carbohydrates. At 12 or 18 months of age, animals were orally administered a single dose of antibiotic cocktail through gavage (250 µL), including ampicillin (1 mg/mL), vancomycin (5 mg/mL), neomycin trisulfate (10 mg/mL), and metronidazole (10 mg/mL). This method of administration and concentrations have been demonstrated to significantly reduce bacterial load within 24 hours post-treatment [14]. After antibiotic treatment, 12-month-old animals received an FMT from a pool of samples collected from untrained donors of the same age (auto-transplant, Auto-Tr) or from young-trained mice (YT-Tr). Animals treated at 12 months underwent a second FMT at 15 months to reinforce the initial treatment and were euthanized at 18 months (Supplementary Fig. 1A). On the other hand, mice treated at 18 months received Auto-Tr and YT-Tr, as well as FMT from young sedentary donors (YS-Tr). These mice did not receive reinforcement treatment and were euthanized at 20 months (Supplementary Fig. 1B). Young male mice (3 months old) were trained on a treadmill at 60% of their peak running speed, which was measured to be 12 m/min in an incremental treadmill test. The training regimen lasted for 1 hour per day, 5 days a week, for 6 weeks. After this training period, fecal samples were collected for FMT preparation (YT-Tr). For Auto-Tr animals, feces were prepared from untrained male donors of the same age as the recipients. For YS-Tr, feces were prepared from untrained 4-month-old male donors. Supplementary Figure 1 depicts the experimental design, including a timeline of the procedures and evaluations performed. All procedures were approved and supervised by the Bioethics Committee of the Faculty of Science at Universidad de Valparaíso (protocol BEA179-22).

### Treadmill Fatigue Test Protocol

Young mice donors were subjected to a treadmill fatigue test to evaluate exercise performance and endurance. Prior to the test, the animals were acclimated to the treadmill over three days at low speeds (10 m/min) for 5, 10, and 15 minutes, respectively. For the fatigue test, the treadmill was set to a slight incline (15°). The test began at a speed of 10 m/min for 30 minutes, after which the treadmill speed was incrementally increased until exhaustion. Exhaustion was defined as the inability to continue running despite gentle encouragement, such as air puffs or soft tapping. Total running distance and maximum speed were recorded for analysis.

### FMT preparation and scheme of administration

Fresh fecal samples from five donor mice per condition were collected in a PBS buffer supplemented with 20% glycerol and 0.05% L-cysteine at a ratio of 1 mL buffer per 100 mg of feces. The samples were homogenized by vortexing, centrifuged at 2000 rpm for 5 minutes at 4°C, and the resulting supernatant was aliquoted into 1 mL tubes and stored at -80°C. Prior to administration, aliquots were thawed at room temperature and used for FMT via oral gavage at a dose of 200 μL per recipient mouse. Twelve-month-old mice underwent FMT following a predefined schedule: (1) an initial antibiotic treatment comprising ampicillin (1 mg/mL), vancomycin (5 mg/mL), neomycin trisulfate (10 mg/mL), and metronidazole (10 mg/mL); (2) one day after completing the antibiotic regimen, FMT was administered daily for three consecutive days during the first week; (3) subsequently, FMT was given once weekly for three weeks. At 15 months of age, reinforcement doses of FMT were administered over three consecutive days without prior antibiotic treatment (see Supplementary Fig. 1A).

All work surfaces and tools were thoroughly cleaned with 70% ethanol before and after each procedure to reduce the risk of contamination. Gavage needles used for FMT administration were sterilized at the start of each procedure, with one needle dedicated to each fecal transplant condition. The same needle was reused for animals within the same group; however, stringent cleaning and handling practices were employed to prevent cross-contamination. The body weight and health status of the mice were monitored throughout the experiment to ensure proper handling and animal welfare. 18-month-old mice followed the same procedure, except they did not receive the reinforcement treatment, see Supplementary Figure 1B.

### Behavioral Tests

To minimize stress-related variables, all animals were acclimatized to the testing room for a minimum of 30 minutes before each behavioral test. This period allowed the mice to adapt to the environmental conditions and reduce potential confounding factors. The behavioral apparatus was meticulously cleaned with 5% ethanol between phases or evaluated mice and with 70% ethanol at the end of each testing day. This rigorous cleaning protocol aimed to eliminate olfactory cues and potential contaminants that may influence mice behavior. For memory-related tests, we used towers and/or columns strategically attached to the platform using a removable tape. This approach was designed to evaluate spatial memory and recognition. The specific positioning of objects aimed to engage mice in memory-dependent tasks. Behavioral tests were recorded using a camera system (model C920, Logitech Co.) at a frame rate of 30 FPS and a resolution of 640x480 pixels. The obtained videos were meticulously analyzed offline using the ANY-maze behavioral software (v.7.09 Stoelting Co., Wood Dale, IL, USA) to ensure accurate and consistent measurements.

#### Open Field

Each mouse was individually placed in the center of a square arena of 40x40 cm surrounded by high walls (30 cm) in a brightly lit room (300 lux). They were allowed free exploration in the open field arena for 5 minutes while filmed by a camera system. Using the software analysis, a series of 10 x 10 cm zones were defined with a total of 16 blocks. The outer zone consisted of 12 blocks while the center zone consisted of 4 blocks (20 x 20 cm). Total distance traveled, average, and maximum speed were evaluated as an indicator of motor activity. Time spent in the center and time in the outer edge were used to assess anxious-like behaviors.

#### Novel Object Recognition (NOR)

**Figure 1.**
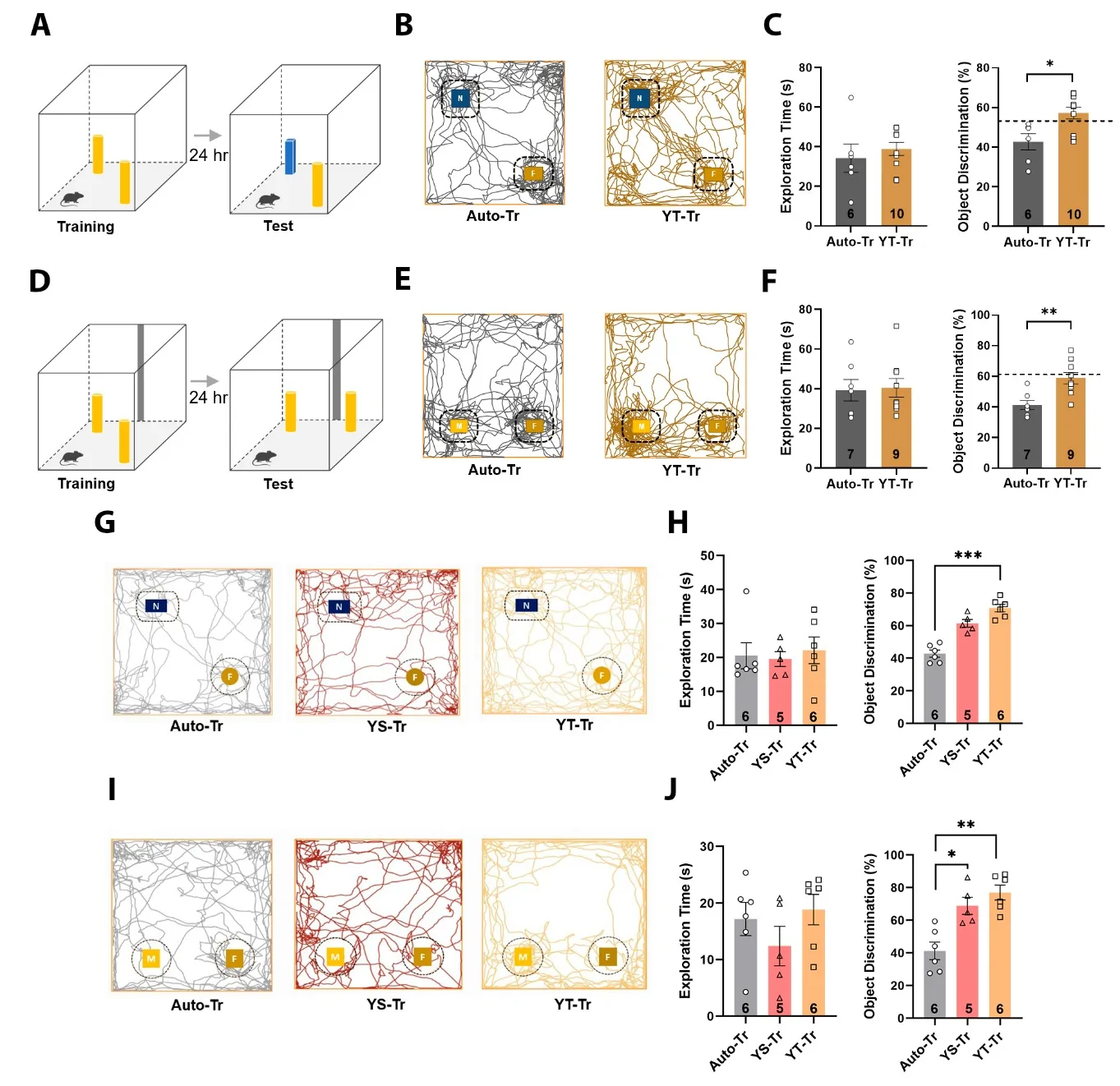
**FMT from young and trained animals improves the performance of aged mice in behavioral tests associated with memory function**. (**A**) A schematic depiction of the novel object recognition test and (B) tracking plot during the novel object recognition test (NOR) for a representative mouse of the Auto-Tr and YT-Tr groups. (**C**) Bar graphs show the total exploration time during the test day (left) and the object discrimination time (right), corresponding to the percentage of the exploration time that the mice spent with the novel object (N=6 for Auto-Tr group and N=10 for YT-Tr). It is observed that the YT-Tr group recognized the novel object more successfully than the Auto-Tr group with a statistical significance. (**D**) Schematic depiction of the object location memory test (OLM) which includes a visual clue as a special reference. (**E**) Representative movement trajectory map during the test day of the OLM test. (**F**) Total exploration time (left) and object discrimination time (right) were also evaluated in the OLM test (N=7 for Auto-Tr group and N=9 for YT-Tr). The YT-Tr group recognized the new location more successfully than the Auto-Tr group with a statistical significance. Points represent data collected from individual mice. Data are presented as means ± SEM; *p < 0.05 and **p < 0.01, as determined by Mann Whitney test. Data for animals treated at 18 months of age are presented in panels (G-J). (**G**) Tracking plots during the novel object recognition (NOR) test for representative mice from the Auto-Tr, YS-Tr, and YT-Tr groups. (**H**) Bar graphs display the total exploration time on the test day (left) and object discrimination time (right), expressed as the percentage of exploration time spent with the novel object (N=6 for the Auto-Tr group, N=5 for YS-Tr, and N=6 for YT-Tr). Only the YT-Tr group achieved statistical significance in novel object recognition compared to the Auto-Tr group. (**I**) Representative movement trajectory maps from the test day of the OLM test. (**J**) Bar graphs illustrate the total exploration time (left) and object discrimination time (right) in the OLM test (N=6 for the Auto-Tr group, N=5 for YS-Tr, and N=6 for YT-Tr). The YT-Tr group outperformed the other groups in recognizing the new location. Points represent data from individual mice. Data are presented as means ± SEM; *p < 0.05, **p < 0.01, ***p < 0.001, as determined by the Kruskal-Wallis test. N= novel object, F= familiar object, M= mobile object.

The task was divided into habituation, training, and test phases. During the initial phase (day 1), the rodent spent 5 minutes in the center of the open field to familiarize itself with the apparatus. Subsequently, the mouse was returned to its home cage. In the training stage the next day, two identical objects were positioned in the apparatus, and the animal had 5 minutes to explore both objects freely. After a 24-hour delay, the test stage took place. In this phase, one of the objects was replaced with a novel one, and the animal had 5 minutes to explore the objects (Fig. 1A). Parameters assessed included the total distance traveled, exploration time for each object, and discrimination index was calculated using the following equation:

$$\mathrm{\% discrimination}=\frac{\mathrm{exploration time in the new localization}}{\mathrm{total exploration time}}x100$$

The exploration time was manually calculated using ANY-maze software, measuring the duration of the animal's nose interaction with the object within a 3 cm range surrounding it. Instances where the animal climbed on or approached the object with body parts other than its head were not considered part of the exploration time.

#### Object Location Memory (OLM)

The animals underwent initial habituation in an empty arena on day 1, with consistent visuospatial cues present in all test stages. A vertically attached black tape served as a cue, positioned at the center of one of the walls. On the subsequent day, two identical objects were introduced into the apparatus at specific locations, allowing the animal to explore them freely for 5 minutes. On the final day, the test stage was conducted, involving the repositioning of one object while the other remained in a fixed location. The mouse was given 5 minutes to freely explore the objects (Fig. 1D). Parameters assessed included the total distance traveled, exploration time for each object, and the discrimination index. Discrimination index was calculated with the following equation:

$$\mathrm{\% discrimination}=\frac{\mathrm{exploration time in the new localization}}{\mathrm{total exploration time}}x100$$

The exploration time was manually calculated using ANY-maze software as the duration of the animal's nose interaction with the object within a 1 cm^2^ range surrounding it. Instances where the animal climbed on or approached the object with body parts other than its head were not considered exploration time.

### LTP evaluation

#### Brain slice preparation

Mice were anesthetized with isoflurane and decapitated. Their brains were then rapidly removed through craniotomy and submerged in cold artificial cerebrospinal fluid (ACSF). The ACSF contained the following (in mM): 124 NaCl, 2.70 KCl, 1.25 KH_2_PO_4_, 1.30 MgSO_4_, 26 NaHCO_3_, 10 Glucose, and 2.50 CaCl_2_. The pH of the ACSF was stabilized at 7.4 by bubbling a mixture of 95% oxygen and 5% CO_2_. Coronal slices of the dorsal hippocampus (300 µm) were cut using a vibratome (Campden Instruments, model MA752, Loughborough, United Kingdom). The slices were then incubated in ACSF for over an hour at room temperature (20-22°C) before being transferred to a 1 mL chamber fixed to a magnifying glass. The slices were continuously perfused with ACSF and maintained at room temperature (21-24°C).

#### Electrophysiological recordings

Field excitatory postsynaptic potentials (fEPSPs) were recorded using a glass pipette (2-4 MΩ, filled with NaCl 1M) positioned in the middle of the stratum radiatum in CA1. The recording electrode was connected to an AC amplifier (P-5 series; Grass) with a gain of 10,000, a low-pass filter of 3.0 kHz, and a high-pass filter of 0.30 Hz. Bipolar cathodic stimulation generated by a Master 8 stimulator (AMPI) was used to activate Schaffer collateral fibers, which were connected to an isolation unit (Isoflex; AMPI, Jerusalem, Israel). The bipolar concentric electrode (platinum/iridium; FHC, Bowdoin, Maine, USA) was placed in the stratum radiatum 100-200 µm away from the recording site. Electric pulses (50 µs and 80-100 µA) were applied to the Schaffer collaterals to record basal excitatory synaptic transmission at 0.1 Hz. Long-term potentiation (LTP) was elicited using a high-frequency stimulation (HFS) protocol. The protocol consisted of two trains of 100 pulses for 1 second (100 Hz), separated by 1 second. HFS was applied after at least 20 minutes of stable baseline recordings. The experiments were conducted at room temperature (20-22°C).

### Gut permeability

#### FITC dextran 4 based method

Ex vivo intestinal permeability was determined using a previously described method with slight modifications [15]. Ileum and proximal colon samples (4-5 cm in length each) were used to make sacs. FITC- Dextran 4 (1 mg/ml) was placed into the intestinal pieces and tied with cotton threads (ileum: 200 μl, colon: 300 μl). The intestinal sacs were placed into 15 mL tube containing Krebs buffer at 37° C and aerated with 95% O2 / 5% CO_2_. Medium was collected at 1-, 2-, and 3-hours points and analyzed for FITC on a fluorescent plate reader at 470 nm (Invitrogen Qubit 4, Thermo Fisher). The apparent permeability of each individual intestinal sac was then calculated. Permeability is expressed as the percentage of FITC concentration outer the sac corrected by tissue area, considering the total amount of FITC charged inside the sac as 100%.

#### Fecal albumin levels

Fecal albumin levels were measured using the Bromocresol Green albumin assay kit (Sigma-Aldrich, MAK124, Saint Louis, MO, USA). Stool samples (100 mg) were diluted in 300 μL of ultrapure water. After centrifugation (1200 RCF, 1 min), the supernatants and the bovine albumin standard were each diluted two-fold in ultrapure water. The manufacturer's instructions were then followed, except that 30 μL of the diluted fecal sample was used to adjust the albumin concentration to a detectable range. After adding 200 μL of BCG reagent and incubating for 5 minutes at room temperature, absorbance at 620 nm was measured, and values were quantified based on the standard curve.

### Western Blot

#### Protein extraction

Tissue sections (approximately 30 mg) of the colon, hippocampus, or muscle were lysed in 300 μl of ice-cold lysis buffer (composition in mmol/L: 1000 Tris-HCl, 500 EDTA, 150 NaCl, 5 sodium deoxycholate, 1 SDS [10%], pH 7.8, NP-40 [10%]) supplemented with a protease inhibitor mixture. The supernatant fractions were recovered by centrifugation at 11,200 g for 10 min at 4°C.

#### Immunoblots

Total protein (30 μg) from each lysate were separated into 6% SDS-polyacrylamide gels (for ZO-1) or 10-16% (for the other identified proteins) and transferred to PDVF membranes (IPVH00010 Millipore, Burlington, MA, USA). Membranes were blocked at room temperature for 1 h in Tris-buffered saline containing 5% PBS, with 0.1% Tween 20. Membranes were then incubated overnight with the following primary antibodies: PSD95 (Rabbit, 1:1000; Cat# 2507, Cell Signaling Technology, Danvers, MA, USA), AMPKα (Mouse, 1:1000; Cat# 2793, Cell Signaling Technology), Phospho-AMPKα (Thr172) (Rabbit, 1:500; Cat# 2531, Cell Signaling Technology), FNDC5/Irisin (Rabbit, 1:1000; Cat# PA5-79279, Thermo Fisher, Waltham, MA, USA), Synaptophysin (Mouse, 1:5000; Cat# 101011, Sysy antibodies, Gӧttingen, Germany), ZO-1 (Rabbit 1:500; Cat# 8193, Cell Signaling Technology) and the loading controls β-tubulin (Mouse, 1:5000; Cat# MA5-16308, Thermo Fisher) and GAPDH (Rabbit, 1:20000; Cat# 2118, Cell Signaling Technology). After primary antibody incubation, membranes were treated with Goat anti-Rabbit IgG HRP secondary antibody (1:10000; Cat# 31460, Thermo Fisher) or Rabbit anti-Mouse IgG HRP secondary antibody (1:10000; Cat# 31450, Thermo Fisher) at room temperature for 90 minutes. Protein bands were visualized using the SuperSignal™ West Pico PLUS chemiluminescent substrate (34580, Thermo Fisher) according to the manufacturer’s instructions.

### Real time PCR

Total RNA was obtained from mice hippocampus employing Trizol (Invitrogen, Corp., Carlsbad, CA, USA) according to the manufacturer’s protocol. cDNA was prepared from 1 μg of RNA, using SuperScript II enzyme (Invitrogen). Quantitative real-time PCR (qPCR) was performed using AriaMx Real-time PCR System (Agilent, Santa Clara, CA, USA) with the SsoAdvanced Universal SYBR Green Supermix (Bio-Rad, Hercules, CA, USA). Expression values were normalized to *Rplp0* and reported in units of 2^-ΔΔCt^ ± SEM. For primer sequences and thermocycling conditions see Supplementary Table 1.

### Immunofluorescence

Colon samples from each mouse were preserved in cassettes in Tissue-Teck (Sakura Finetek U.S.A, Torrance, CA) at -80°C. Briefly, 16 µm frozen sections of mouse colon were fixed in acetone/metanol 50% and immunostained using anti-ZO-1 (Cat# 33-9100 mouse monoclonal, Invitrogen), followed by Alexa Fluor 488-conjugated secondary antibody (Cat# A11001, Invitrogen). Nuclei were stained with DAPI. Stained sections were imaged with a X40 water immersion objective on a confocal microscope 710 Zeiss with continuous and adjustable spectral scanning system. Quantification was performed by calculating the ZO-1-positive area using ImageJ software, with the Auto-Tr group serving as the control. A secondary antibody control was included to eliminate the possibility of nonspecific fluorescence.

### ELISA multiplex

Serum samples obtained from mice were utilized to quantify thirteen specific cytokines through Q-Plex Chemiluminescent ELISA (Quansys Bioscience, Logan, UT, USA) in accordance with the manufacturer’s guidelines. The levels of Granulocyte-macrophage colony-stimulating factor (GM-CSF), IL-1α, IL-1β, IL-2, IL-3, IL-4, IL-6, IL-10, IL-12p70, IL-17A, Macrophage Inflammatory Protein-1 Alpha (MIP-1α), RANTES (also known as Chemokine (C-C motif) ligand 5), and TNFα were assessed using a Bio-Rad Chemi-Doc camera (Bio-Rad, Hercules, CA, USA) and Q-View Software (Quansys Bioscience). The concentration of cytokines is presented as pg/mL.

### Short-chain fat acid analysis

#### SCFA extraction in feces

After mice euthanasia cecal content was collected for SCFAs quantification. Three fecal samples per animal were processed independently in triplicate. A total 10 mice were included for SCFA characterization. For fecal SCFAs extraction, 0.4 gram of frozen feces (-80° C) were thawed in ice and suspended in 1.5 mL of distilled water, homogenized (manually with a plumber), vortexed for 1 min and finally, sonicated in an ice bath for 30 minutes. Samples were then centrifuged at 9000x*g* for 30 minutes at 4°C and 0.1 mL of suspension was collected in a new 1.5 mL plastic tube. Ten microliters of tropic acid 6 mM (Internal standard) and 1 µL of HCl were added to the suspension to adjust the pH to 2. Diethyl ether was then added, vortexed for 30 seconds and decanted for 2 min to allow the separation of aqueous and organic phases; then the upper phase (organic) was collected in a new tube. The step of diethyl ether extraction was repeated three more times, collecting all the organic phases together in the same tube. A pinch of NaSO_4_ was added to the organic acids fraction to remove any possible trace of water. To obtain the derivatized SCFAs, 200 µL of dehydrated organic phase was transferred into a glass vial, and then 10 µL of N,O-Bis(trimethylsilyl)trifluoroacetamide (BSTFA) were added and incubated for 1hr at 37° C. One microliter was injected in Gas Chromatography Mass Spectrometry (GC/MS).

#### GC/MS analysis

Chromatographic analysis was carried out using an Agilent GC system coupled with a mass spectrometer detector (GCMSD 7890A/5975, Agilent, USA) and supported with a fused silica column Rxi-5ms (30m, 0.25mm ID, 0.25µm, Restek. USA). The separation of SCFAs was carried out with a flow rate of helium of 1mL/min and a temperature ramp that initiated at 40° C, maintained for 2 minutes, raised to 150° C at 15° C/minute, held for 1 minute, and increased to 300° C at 30°C/minute, and finally held to 300° C for 5 minutes. The total run time was 20 minutes. Inlet and auxiliary temperatures were 260° C and 280° C, respectively. One microliter from the derivatized sample was injected in split mode (1:25). Solvent delay was set up to 3 minutes and mass detection was obtained using full scan mode in the range of 50- 480 *m/z* with an ionization energy in electro impact mode of 70 mV. Compound identification was validated by injecting pure standards and comparing retention times and corresponding MS spectra. Selected ion monitoring (SIM) mode was used for quantification, utilizing the target ion, and confirming with qualitative ions. Specifically, the target ion (*m/z*) for acetic, propionic, butyric, valeric, and tropic (internal standard) acids were 117, 131, 145, 159, and 280, respectively. The quantification of the abundance (micromoles) of each short-chain fatty acid (SCFA) in every independent sample was automatically performed by interpolating their relative abundance on a daily freshly prepared calibration curve. This curve was created using the Chemstation MSD Data Analysis software from Agilent, USA.

### Microbial DNA extraction and sequencing

Total microbial DNA extraction was performed from 180-220 mg of feces homogenized in 1 ml of InhibitEX Buffer and the manufacturer's instructions were followed (QIAamp Fast DNA Stool Mini Kit (Qiagen, Maryland, USA). The amplification of hypervariable regions of V3-V4 16S genes was performed using 357F and 806R primer pairs. PCR reactions were performed in Taq buffer 1X final concentration, 2 nM of MgCl2, 0.3 nM of dNTPs, 0.3 μM of each primer, 2.5 units of GoTaq Flexi DNA Polymerase (Promega, Madison, WI, USA) and 4 ng of template DNA. Amplification conditions were 3 min of initial denaturation at 94°C, 28 cycles of 94°C for 30 s, 57°C for 1 min and 72°C for 1.5 min, followed by a final extension of 72°C for 10 min. Illumina primer constructs were obtained from Earth Microbiome Project. Combined amplicons were quantified using a standard qPCR assay using a Kapa Library Quant Kit Illumina (Roche, Basel, Switzerland) according to manufacturer instructions. The amplified library was analyzed in Bioanalyzer 2100 (Agilent Technologies, California, USA) using a DNA 1000 chip (Agilent Technologies, California, USA) as per the manufacturer’s instructions. The library was then loaded onto the Illumina MiSeq platform for cluster generation and subjected to paired-end Sequencing.

### Gut microbiome data analysis

Bioinformatics analysis was performed with QIIME 2 v2022.2. Raw sequences of the paired reads were demultiplexed and quality filter with q2-demux plugin followed by denoising with DADA2 amplicon sequence variants (ASVs). The taxonomy of the 16S rRNA ASVs was assigned using the scikit-learn plugin with a naive Bayes taxonomy classifier training on Silva 138 database. ASVs that were taxonomically annotated as “Mitochondria” and “Chloroplast” were excluded from the subsequent bacterial community analysis.

Alpha diversity metrics were calculated using the microbiome v1.24 R package and were compared between groups using Wilcoxon test. p-value < 0.05 was considered as statistically significant. Beta diversity was examined using a multidimensional scaling (MDS) with a Bray-Curtis dissimilarity metric, using phyloseq v1.46 package. Differences in the samples’ beta diversity were measured using the permutational multivariate analysis of variance (permANOVA) and multivariate homogeneity of groups dispersions, allowing for 999 permutations, using vegan v2.6 package for R.

To identify differential abundant taxa, we performed a DESeq2 analysis with only the ASVs present in at least three of the replicates, using the microbiome Marker v1.8 R package. Briefly, to assess differential abundance of taxa, DESeq2 assumes a negative binomial distribution for read counts. A null hypothesis that states a set of common parameters for the binomial negative distribution across taxa is tested, with an alternative hypothesis that states different parameters are needed to model the distribution. We use PICRUSt2 for predicting functions inferred from 16S rRNA sequences. We collected the results of PICRUSt2 predictions based on different sources of gene family databases, including KEGG orthologs (KOs), Enzyme Commission numbers (EC numbers), and MetaCyc pathway. Graphics were constructed using ggplot2 v3.5.1 (Wickham, 2009), ggpubr v0.6, Enhanced Volcano v1.2, and microViz v0.12.1 R packages. All calculations were performed in R v4.3.3 environment.

### Statistical analysis

This study employed blinding for several analyses, including electrophysiological recordings, ELISA multiplex assays, ZO-1 level sample preparation, SCFA evaluations, and gut microbiota composition studies. Outliers were identified and removed based on a threshold of ±2 standard deviations from the mean. Our animals shared key characteristics, such as body weight and fragility scores, indicating a similar health status. Based on this, group assignment was performed randomly. The data are expressed as mean±SEM. The Mann-Whitney U test and the Kruskal-Wallis test, along with Dunn’s post-hoc analysis for multiple comparisons, were employed to identify significant differences between groups. These non-parametric methods are suitable for comparing two or more independent groups when the data do not follow a normal distribution. These tests were conducted using a two-tailed criterion, with statistical significance set at p < 0.05. Statistical analysis was performed using GraphPad Prism 8 software (GraphPad, San Diego, CA, USA).

**Figure 2.**
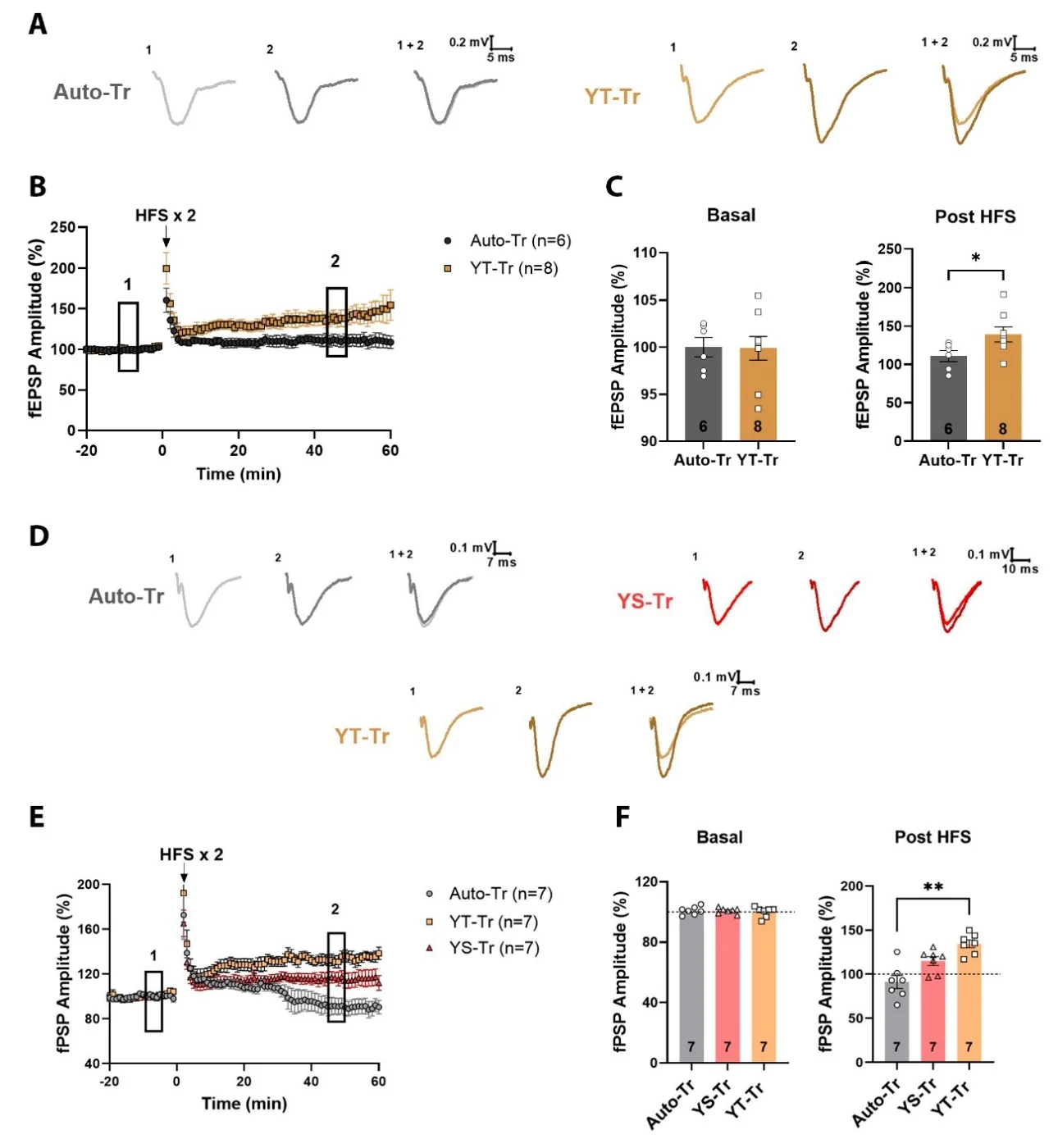
**FMT from young-trained donors restored the ability to induce Long-Term Potentiation (LTP) in the CA1 hippocampus of aged mice**. (**A**) Representative traces of fEPSP obtained before (1) and after (2) the HFS protocol in Auto-Tr (left) and YT-Tr (right) samples from animals treated at 12 months of age. (**B**) Time course of normalized fEPSP amplitude during LTP recording in CA3-CA1 synapses in hippocampal slices from both groups of animals treated at middle age. (**C**) Bar graphs showing the average percentage of fEPSP amplitude corresponding to time 1 (Basal) and time 2 (Post-HFS) in animals treated at 12 months of age (N = 4, one or two brain slices per mouse; n = 6-8). Hippocampal neurons from YT-Tr mice displayed an LTP response after stimulation, a process not observed in the Auto-Tr group. Data are presented as means ± SEM; *p < 0.05, as determined by Mann-Whitney test. Data for animals treated at 18 months of age are presented in (D-F). (**D**) Representative traces of fEPSP obtained before (1) and after (2) the HFS protocol in Auto-Tr, YS-Tr, and YT-Tr (right) samples from animals treated at 18 months of age. (**E**) Time course of normalized fEPSP amplitude during LTP recording in CA3-CA1 synapses in hippocampal slices from the three groups of animals treated at 18 months of age. (**F**) Bar graphs showing the average percentage of fEPSP amplitude corresponding to time 1 (Basal) and time 2 (Post-HFS) in animals treated at 18 months of age (N = 4, one or two brain slices per mouse; n = 7). Hippocampal neurons from YT-Tr mice displayed a significantly increased LTP response after stimulation compared to the Auto-Tr group. YS-Tr group did not reach significance. Data are presented as means ± SEM; **p < 0.01, as determined by Kruskal-Wallis test.

## RESULTS

### Exercise induces physiological and gut microbiota adaptations in young mice

Three-month-old male mice were trained on a treadmill for 1 hour per day, 5 days per week, for 6 weeks at 60% of their maximal running speed. Following this training regimen, we assessed fatigue resistance in the mice. Results showed that physically trained mice ran for significantly longer durations and distances compared to untrained controls (Supplementary Fig. 2A-B). We also examined AMPK phosphorylation (Thr172) levels in the gastrocnemius and tibialis anterior muscles, a well-established biomarker of exercise-induced muscle adaptation [16]. Analysis revealed that AMPK phosphorylation was markedly increased in trained mice compared to sedentary counterparts (Supplementary Fig. 2C). Lastly, we evaluated the gut microbiota composition in trained mice compared to untrained controls, finding no differences in alpha diversity between the groups (Supplementary Fig. 3A). However, beta diversity, as evaluated by Bray-Curtis dissimilarity, revealed distinct bacterial community compositions between trained and sedentary mice (permANOVA p=0.033) (Supplementary Fig. 3B). Additionally, we observed changes in the abundance at the genus level, with *Muribaculaceae*, *Alistipes*, and *Akkermansia* being enriched in the gut microbiota of trained young mice (Supplementary Fig. 3C). These data demonstrate that this treadmill exercise routine induces functional and biochemical adaptations in mouse muscles, while also promoting changes in gut microbiota composition.

### FMT from young, trained donors enhances cognitive function and synaptic plasticity while reducing neuroinflammation in aged mice.

After antibiotic treatment, 12-month-old male mice received a fecal FMT using pooled samples from either age-matched donors (referred to as auto-transplant or Auto-Tr) or young mice that had undergone treadmill training (YT-Tr). At 15 months old, the mice received a second FMT as a reinforcement of the initial transplant, again using fecal samples from either age-matched older donors (Auto-Tr) or young trained donors (Supplementary Fig. 1A). One month after FMT reinforcement, we applied behavioral tests closely aligned with hippocampal functioning to assess cognitive performance in aged mice, specifically the novel object recognition test (NOR) and the object location memory test (OLM). In the NOR test, which evaluates recognition memory, the Auto-Tr group, comprised of aged mice receiving FMT from age-matched donors, demonstrated a discrimination index of 42.73 ± 4.11%. In contrast, the YT-Tr group, consisting of aged mice who previously received FMT from young-trained donors, exhibited significantly superior performance with an index of 57.24 ± 3.03% (Fig. 1B-C). This difference suggests that FMT from young-trained donors enhances the ability of aged mice to recognize and remember novel objects, a key aspect of cognitive function. This finding is important as recognition memory deficits are common in aging and neurodegenerative diseases [17].

Moving to the OLM test, which assesses spatial memory, the Auto-Tr group allocated 41.24 ± 2.92% of exploration time to the mobile object. Conversely, the YT-Tr group displayed a significantly elevated discrimination index of 58.87 ± 3.73% (Fig. 1E-F). This improvement in spatial memory suggests that FMT from young-trained donors positively impacts the ability of aged mice to remember object locations in space. Spatial memory deficits are also prevalent in aging and neurodegenerative conditions [17]. In summary, these results demonstrate that aged mice (16 months old) receiving FMT from young-trained donors exhibit notable improvements in cognitive performance compared to those receiving FMT from age-matched donors. We also evaluated locomotion activity (Supplementary Fig. 4A-D) and anxious behavior (Supplementary Fig. 4E-F) in the open field test, finding no differences between the groups. To investigate the impact of exercise on our findings, we conducted NOR and OLM tests in a separate group of aged mice. These mice received FMT at 18 months from three types of donors: age-matched sedentary donors (Auto-Tr), young sedentary donors (YS-Tr), and young trained donors (YT-Tr) (Supplementary Fig. 1B). We observed that, in both tests, when comparing YS-Tr and YT-Tr to Auto-Tr groups, aged mice receiving gut microbiota from young donors showed improved cognitive performance, especially when the young donor was trained. For NOR test the Auto-Tr group had a discrimination index of 42.84 ± 2.20%, meanwhile YS-Tr had 61.40 ± 2.36% and YT-Tr 70.80 ± 2.35% (Fig. 1G-H). For OLM evaluation, Auto-Tr mice displayed 41.23 ± 5.37% of exploration time for the mobile object, YS-Tr and YT-Tr had 68.86 ± 5.24% and 76.99 ± 4.52% respectively (Fig. I-J).

Given that synaptic plasticity is widely recognized as the main process of learning and memory, with alterations in hippocampal plasticity closely associated with cognitive impairments [18], we sought to study the mechanisms underlying these cognitive enhancements. To this end, we evaluated long-term potentiation (LTP) at CA3-CA1 synapses in brain slices obtained from both the Auto-Tr and YT-Tr groups at 18 months of age. Synaptic plasticity, particularly LTP, is a critical indicator of the brain's ability to adapt and encode memories [18]. To evaluate synaptic plasticity, we started with a 20-minute baseline recording of field excitatory postsynaptic potentials (fEPSPs) in the CA1 region of the hippocampus. Following this baseline assessment, we administered two trains of high-frequency stimulation (HFS) protocol, each consisting of stimuli at 100 Hz for 1 second. The average normalized percentage of the fEPSP amplitude between 45-50 minutes post-HFS was 111.1 ± 7.14% in the Auto-Tr group, whereas the fEPSP amplitude from YT-Tr group exhibited a higher value reach a 139.2 ± 9.74% (Fig. 2A-C). This difference underscores the impact of FMT from young-trained donors on synaptic plasticity, with the YT-Tr group demonstrating significantly enhanced LTP compared to their counterparts in the Auto-Tr group. We also evaluated the LTP response in animals that received FMT at 18 months of age, including those with YS donors. Both the YS-Tr and YT-Tr groups exhibited increased fEPSP amplitudes compared to the Auto-Tr group, with the YT-Tr group showing significantly greater increments. The average normalized percentage of the fEPSP amplitude post-HFS was 91.1 ± 7.27% in the Auto-Tr group, and 114.9 ± 4.95% and 134.4 ± 4.36% for YS-Tr and YT-Tr respectively (Fig. 2D-F). These data indicate that gut microbiota from young, trained donors have a more substantial impact on the cognitive function of older mice compared to microbiota from young, sedentary donors.

**Figure 3.**
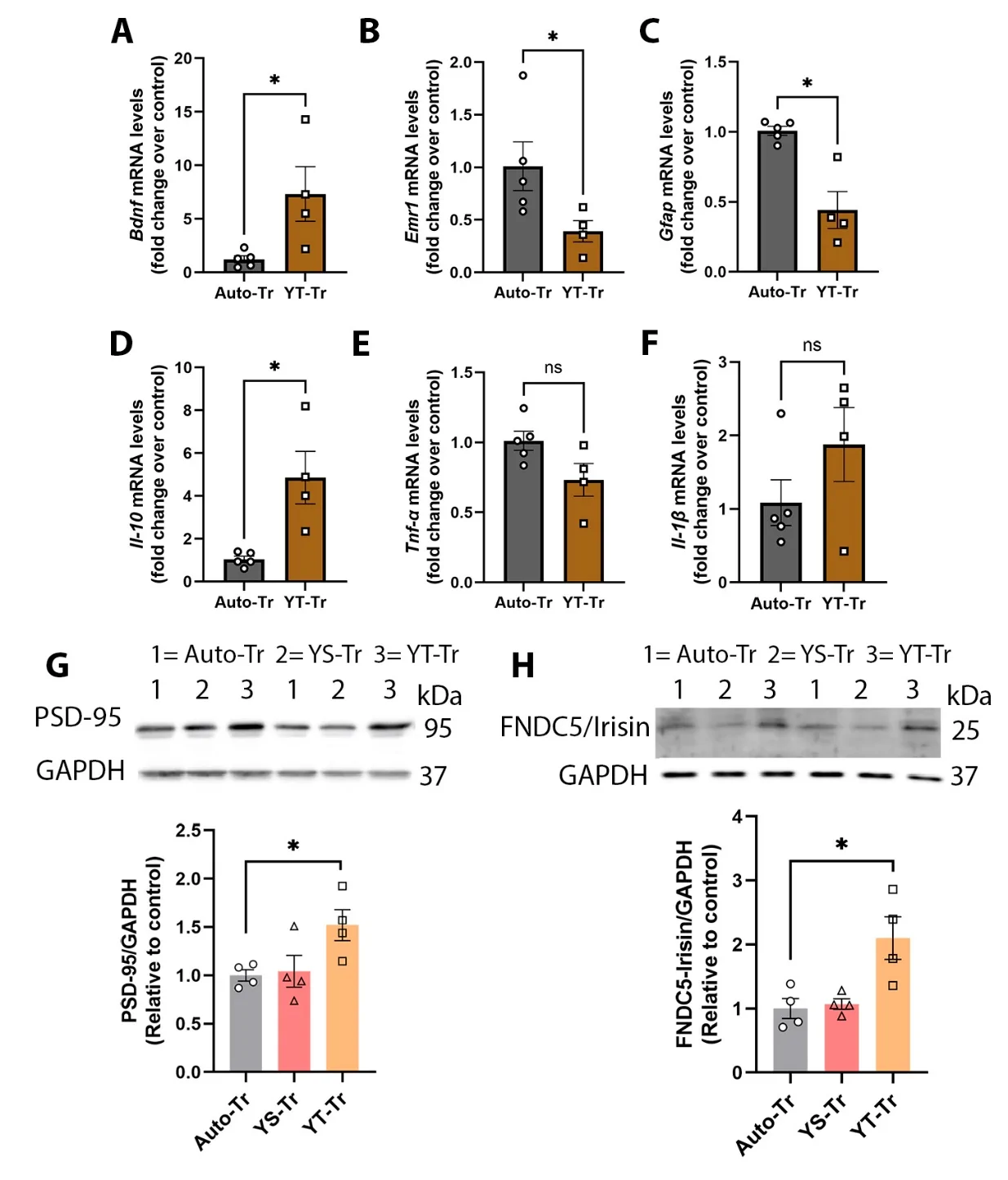
**FMT from young-trained donors reduces neuroinflammation and enhances the levels of synaptic plasticity modulators in the hippocampus of aged mice**. mRNA levels of (A) *Bdnf*, (B) *Emr1*, (C) *Gfap*, (D) *Il-10*, (E) *Tnf-α*, and (F) *Il-1β* were measured by qPCR in hippocampal samples from Auto-Tr (N = 5) and YT-Tr (N = 4) mice that received FMT at middle age. Neuroinflammatory markers, such as *Emr1* and *Gfap*, showed reduced mRNA levels in animals that received young-trained gut microbiota. Conversely, mRNA levels of the anti-inflammatory cytokine *Il-10* and the synaptic plasticity regulator *Bdnf* were elevated in the hippocampus of YT-Tr mice. Data are presented as means ± SEM; *p < 0.05, as determined by the Mann-Whitney test. Protein levels of synaptic plasticity modulators, PSD-95 (95 kDa) (G) and FNDC5/Irisin (25 kDa) (H), were measured by immunoblot in the hippocampus of Auto-Tr, YS-Tr, and YT-Tr mice (N = 4) that received FMT at 18 months of age. GAPDH (37 kDa) was used as a control for total protein content. PSD-95 and FNDC5/Irisin levels were significantly increased in the hippocampal tissue of YT-Tr mice compared to the Auto-Tr and YS-Tr groups. Data are presented as means ± SEM; *p < 0.05, as determined by the Kruskal-Wallis test.

Additionally, we evaluated gene expression in hippocampal samples from 18-month-old mice treated with FMT during middle age. In the YT-Tr group, we observed increased mRNA levels of brain-derived neurotrophic factor (*Bdnf*) (Fig. 3A), a well-known modulator of synaptic plasticity in the hippocampus [19]. We detected reduced mRNA levels of *Emr1* (EGF-like module-containing mucin-like hormone receptor-like, also known as F4/80) (Fig. 3B), a marker of microglial activation [20], and *Gfap* (Glial fibrillary acidic protein) (Fig. 3C), a marker of astrocyte activation [21]. The YT-Tr group also exhibited increased mRNA levels of neuroprotective cytokine *Il-10* [22] compared to the Auto-Tr group (Fig. 3D). However, no significant changes were observed in the mRNA levels of *Tnf-α* or *Il-1β* between the groups (Fig. 3E-F). At the protein level, we observed higher levels of PSD95 (Postsynaptic density protein 95) (Fig. 3G), a pivotal postsynaptic scaffolding protein in excitatory neurons [23], and FNDC5 (Fibronectin type III domain-containing protein 5)/Irisin (Fig. 3H), a critical regulator of cognitive function associated with exercise [24], in the hippocampus of the YT-Tr group compared to both the YS-Tr and Auto-Tr groups in animals that received FMT at 18 months of age. Meanwhile, Synaptophysin (SYP), a presynaptic regulator of synaptic plasticity [25] showed higher levels in both the YS-Tr and YT-Tr groups compared to the Auto-Tr group (Supplementary Fig. 5). These findings suggest that FMT from YT donors improves cognition in aged mice by modulating proteins involved in the homeostasis of synaptic plasticity and reducing neuroinflammation in the hippocampus.

### FMT from Young-Trained Donors Reduces Gut Permeability and Increases ZO-1 Levels in the Colon of Aged Mice.

The intricate interplay between the gut microbiota and intestinal barrier function has gained significant attention in recent years, particularly concerning its implications for overall health. Growing evidence suggests that the composition and diversity of the gut microbial communities play a crucial role in regulating intestinal permeability, which in turn influences the paracellular passive transport of nutrients, microbial metabolites, and potentially harmful substances across the gut epithelium [26]. The impact of chronic exercise on gut microbiota composition is particularly interesting due to its subsequent effects on intestinal barrier integrity. This exercise-induced modulation of the gut microbiota holds promise in strengthening intestinal barrier function and mitigating the translocation of pro-inflammatory molecules, such as lipopolysaccharides (LPS), into systemic circulation [27]. Moreover, research has demonstrated the positive effects of regulating intestinal permeability on cognition and central nervous system (CNS) function [28].

**Figure 4.**
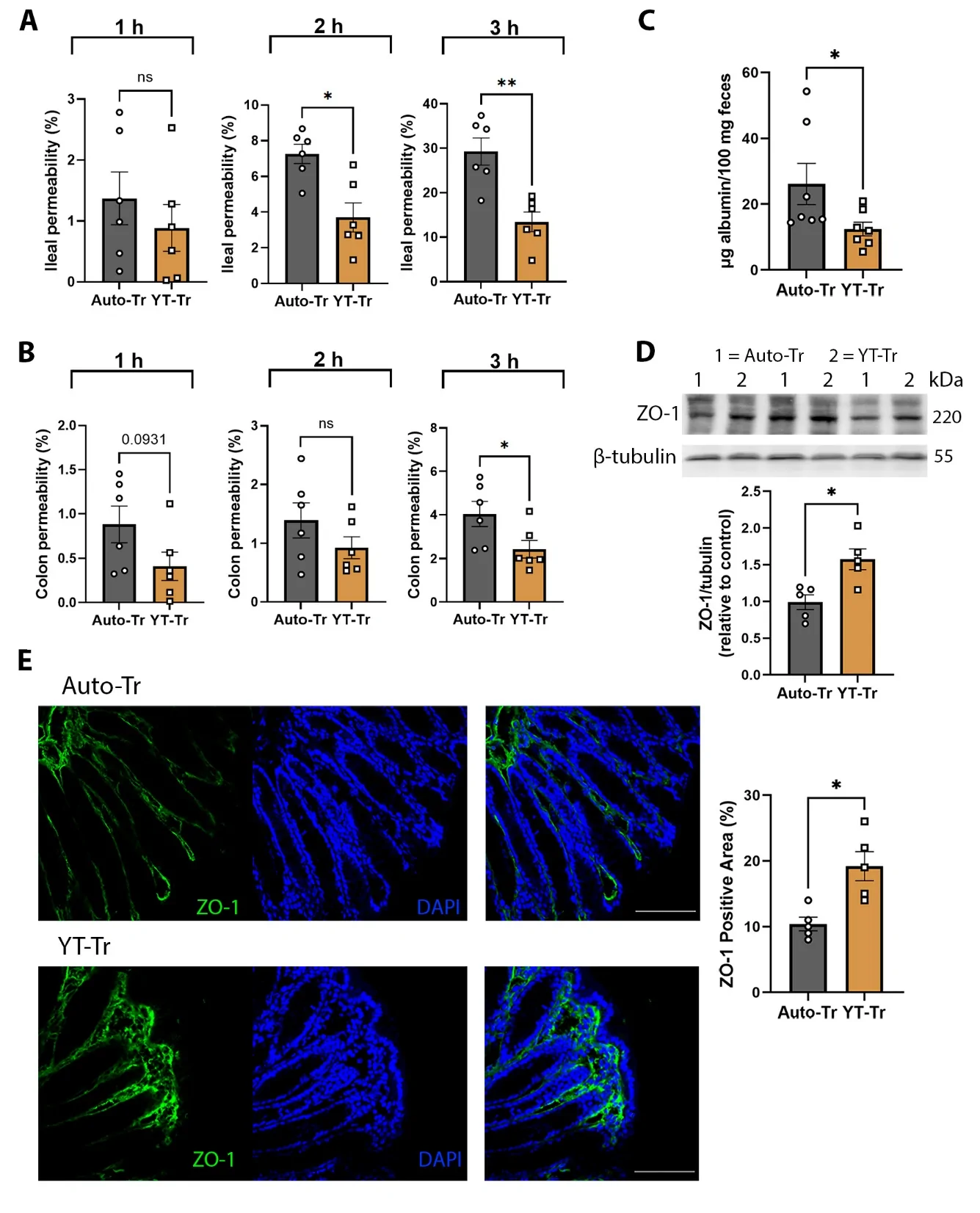
**FMT from young-trained donors reduced ileal and colonic permeability and increased colonic ZO-1 levels in aged mice**. Intestinal sacs were prepared from the ileum (A) and proximal colon (B) of Auto-Tr and YT-Tr mice (N=6). The sacs were loaded with a 2 mg/ml solution of FITC-Dextran 4 (MW 4,400 Da), and the flux of the FITC-Dextran permeability marker was measured at 1, 2, and 3 hours after loading. Ileal permeability was reduced in the YT-Tr group at 2 and 3 h after the assay began, compared to the Auto-Tr group. Colonic permeability was reduced in the YT-Tr group at 3 h after FITC-Dextran 4 loading, compared to the Auto-Tr group. Permeability is expressed as the percentage of FITC concentration outside the sac, corrected by tissue area, with the total amount of FITC inside the sac considered as 100%. (**C**) Fecal albumin concentrations were measured using the Bromocresol Green assay (N=7). Albumin levels were significantly lower in stool samples of YT-Tr 18-month-old mice compared to Auto-Tr old mice. (**D**) ZO-1 (220 kDa) protein levels in mouse colon sections were measured by immunoblot. β-Tubulin (55 kDa) was used as a control for total protein content. ZO-1 levels were increased in colon sections of YT-Tr old mice compared to the Auto-Tr group (n=5). (**E**) On the left, representative immunostainings for ZO-1 (green) are shown in 16 µm frozen sections of mouse colon from 18-month-old Auto-Tr and YT-Tr mice. In the center, nuclei stained with DAPI are displayed. On the right, a composite image is presented. Scale bars represent 100 μm. The bar graph shows an increased positive area for ZO-1 in colonic samples of YT-Tr mice compared to Auto-Tr (N=5). All data are presented as means ± SEM; *p < 0.05 and **p < 0.01, as determined by Mann Whitney test.

In this context, we evaluated intestinal permeability in aged mice subjected to FMT in middle age from young-trained donors. We found that 18-month-old animals who received FMT from young-trained donors exhibited a significant reduction in ileal permeability compared to those from the Auto-Tr group. This reduction was observed at two and three hours from the beginning of the FITC assay (Fig. 4A). Additionally, the colonic intestine also exhibited reduced permeability in the YT-Tr group compared to the Auto-Tr animals, with significance observed three hours after the test started (Fig. 4B). We also evaluated intestinal permeability by measuring albumin levels in stool samples. We found a significant reduction in fecal albumin in the YT-Tr group (12.5±2.1 mg per 100 mg feces) compared to the Auto-Tr group (26.1±6.2 mg per 100 mg feces) (Fig. 4C). Since intestinal permeability is strongly associated with the levels of the tight junction protein zonula occludens-1 (ZO-1) [26], we studied ZO-1 levels using western blot and immunofluorescence in colonic samples. We found higher levels (Fig. 4D) and increased positive area (Fig. 4E) of ZO-1 in the colon of YT-Tr mice compared to the Auto-Tr group.

### FMT from Young-Trained Donors Decreases Inflammatory markers in the blood of Aged Mice.

A reduction in intestinal permeability may be associated with a reduction of inflammaging [26], a process characterized by chronic low-grade inflammation observed in aged individuals [2]. Inflammaging has been implicated in the pathogenesis of many age-related diseases, including neurodegenerative disorders such as Alzheimer's disease (AD) [2]. Emerging evidence suggests that compromised gut barrier function and increased intestinal permeability contribute to inflammaging by facilitating the translocation of microbial products, such as LPS, flagellin, or others, into systemic circulation, thereby triggering systemic inflammation [3].

**Figure 5.**
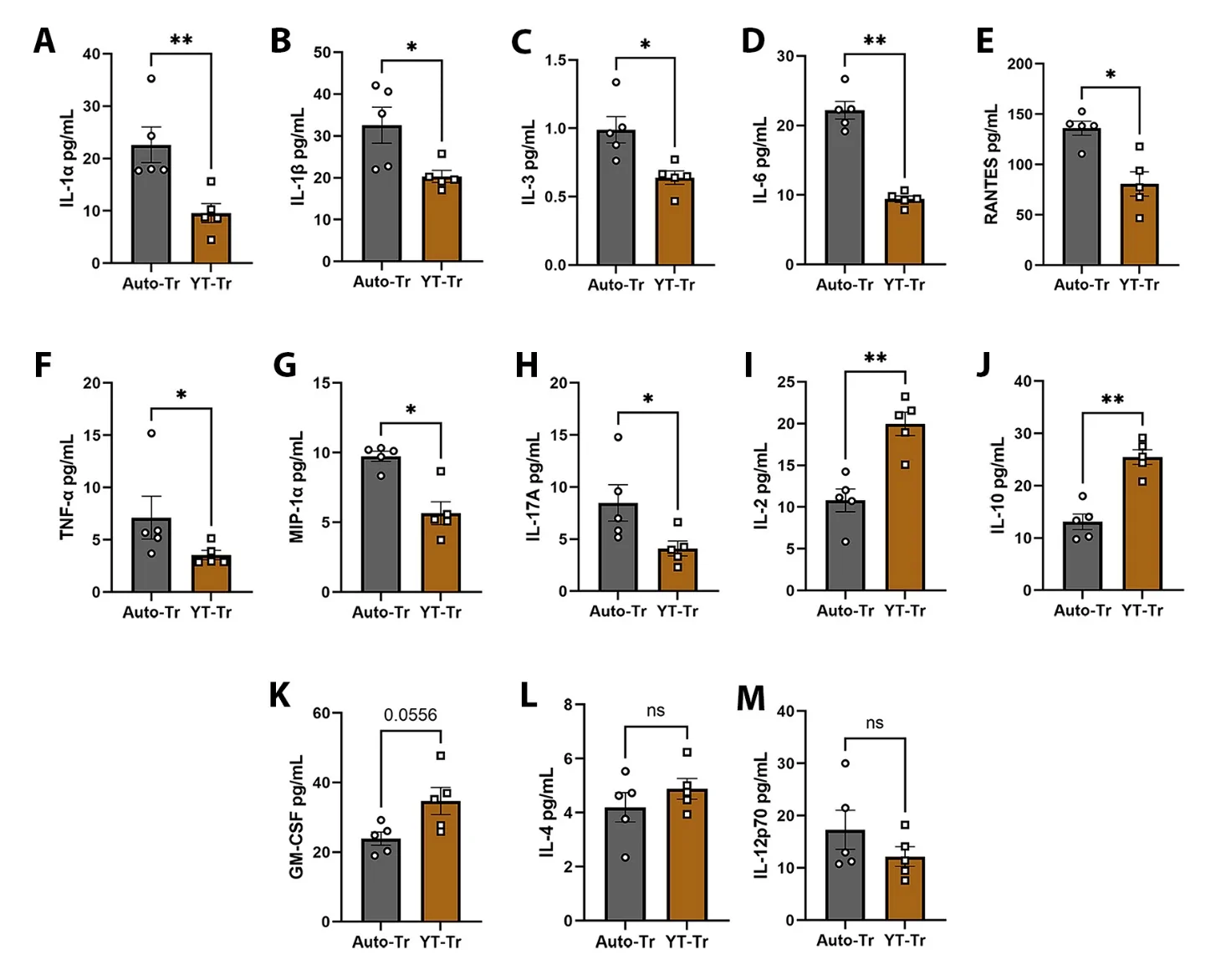
**FMT from young-trained donors modulates inflammation-related markers levels in the serum of aged mice**. 13 inflammation-related factors were evaluated in the serum of Auto-Tr and YT-Tr groups by ELISA-plex assay. (**A-H**) Serum concentrations of pro- inflammatory factors, IL-1α, IL-1β, IL-3, IL-6, RANTES, TNF-α, MIP-1α, and IL-17A, were reduced in the YT-Tr animals compared to the Auto-Tr group. Meanwhile cytokines with anti-inflammatory or with positive cognitive effects actions (IL-2, IL-10, GM-CSF) showed higher serum levels in YT-Tr mice than Auto-Tr ones (I-K). Concentration is expressed as pg/mL. Data are presented as means ± SEM, N=5. *p < 0.05 and **p < 0.01 as determined by Mann Whitney test.

Consequently, interventions to improve the gut barrier integrity and reduce intestinal permeability could mitigate inflammaging and its detrimental effects on cognitive function during aging. We evaluated protein levels of several inflammatory markers such as GM-CSF, IL-1α, IL-1β, IL-2, IL-3, IL-4, IL-6, IL-10, IL-12p70, IL-17A, MIP-1α, RANTES and TNFα in the serum of Auto-Tr and YT-Tr aged mice. We found reduced serum levels of IL-1α, IL-1β, IL-3, IL-6, RANTES, TNF-α, MIP-1α, and IL-17A in YT-Tr 18 months old mice age compared to aged mice from the Auto-Tr group (both groups subjected to FMT in middle age) (Fig. 5A-H). Cytokines associated with anti-inflammatory and positive cognitive effects such as IL-2, and IL-10 [29, 30] were found elevated in the serum of YT-Tr animals compared to Auto-Tr (Fig. 5I and 5J). GM-CSF showed a tendency to increase in YT-Tr mice as well (p= 0.0556) (Fig 5K). No changes were observed for serum levels of IL-4 and IL-12p70 between groups (Fig. 5L and 5M).

### FMT from Young-Trained Donors increases SCFA Levels in the Cecal Content of Aged Mice.

**Figure 6.**
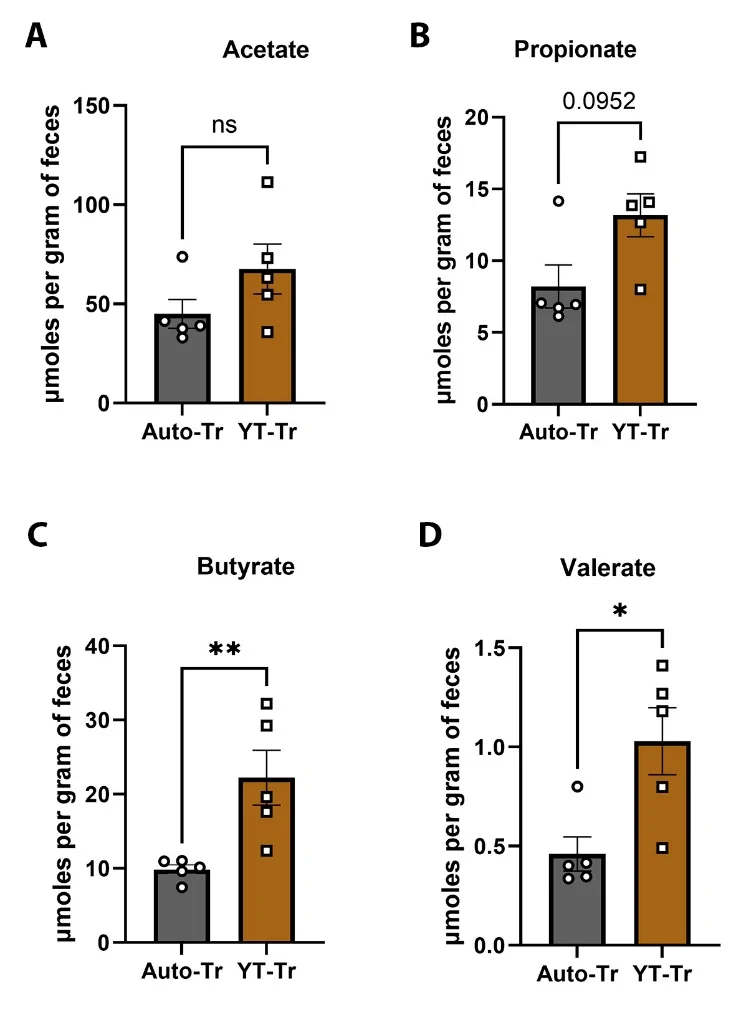
**FMT from young-trained donors increased levels of SCFA in the cecal content of aged mice**. Cecal content concentrations of (A) acetate, (B) propionate, (C) butyrate, and (D) valerate in 18-month-old mice from Auto-Tr and YT-Tr groups. Butyrate and valerate levels were significantly higher in the YT-Tr group compared to the Auto-Tr group. Data are presented as means (µmoles/gram of feces) ± SEM, N=5. *p < 0.05 and **p < 0.01 as determined by Mann Whitney test.

It has been proposed that the intestinal microbiota can modulate gut permeability through the production of short-chain fatty acids (SCFAs), with evidence suggesting that these compounds indeed possess the capacity to decrease intestinal permeability [31]. Specifically, acetate, propionate, valerate, and butyrate have been implicated in the maintenance of gut barrier integrity [31]. Studies have elucidated that SCFAs can enhance the expression of tight junction proteins, including occludin and ZO-1, thus promoting epithelial barrier function [31]. Moreover, the production of SCFAs has been associated with anti-inflammatory effects during aging, attenuating age-related intestinal inflammation and preserving gut homeostasis [5]. Emerging evidence suggests a link between SCFA production and cognitive function in older adults, with higher levels of SCFAs correlating with improved cognitive performance and reduced risk of cognitive decline [32]. These findings underscore the potential role of SCFAs as key mediators in gut-brain communication and highlight their therapeutic potential in mitigating age-related intestinal dysfunction and cognitive decline. We assessed acetate, propionate, butyrate, and valerate concentration in the cecal content of 18-month-old mice subjected to FMT in middle age. We observed that animals receiving FMT in the YT-tr group exhibited significantly higher levels of butyrate and valerate compared to the Auto-Tr group (Fig. 6C-D). Acetate levels did not change between groups (Fig. 6A), and although higher levels of propionate were observed in YT-tr animals, the difference did not reach statistical significance (p=0.0952) (Fig. 6B).

### FMT from Young-Trained Donors Increased Representation of Bacteria Associated to Beneficial Effects on Cognitive Function in Aged Mice.

**Figure 7.**
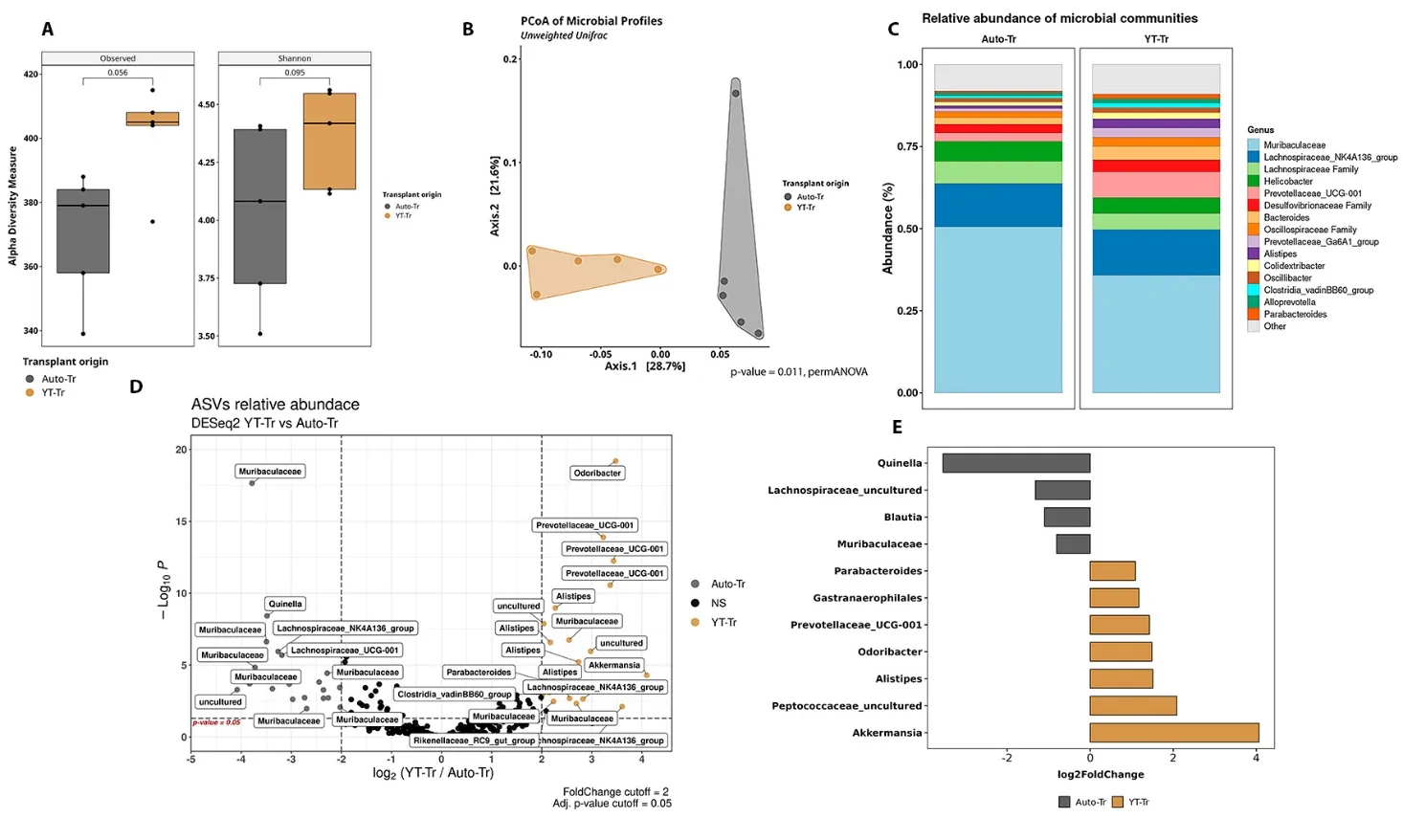
**The gut microbiome of YT-Tr mice has a different composition compared to Auto-Tr mice’s gut microbiome**. (**A**) On the left side, the observed count of enriched ASVs is compared between the Auto-Tr (N=5) and the YT-tr (N = 5) group. The data is displayed as the precise number of ASVs. On the right side, the Shannon index is examined in both the Auto-Tr (N=5) group and the YT-tr (N = 5) group. There is a tendency for increased diversity in the YT-Tr group. (**B**) PCoA of beta-diversity of Auto-Tr (N = 5) and YT-Tr (N = 5) as measured by Unweighted UniFrac distance. The bacterial communities in Auto-Tr and YT-Tr are dissimilar to each other, permANOVA p=0.011. (**C**) Mean relative abundance at genus level among groups. Genera with a relative abundance below 1% are grouped together and denoted as "others." (D) Volcano plot comparing Auto-Tr (N = 5) and YT-Tr (N = 5), highlighting significantly enriched bacterial amplicon sequence variants (ASVs) (P < 0.05) identified by DEseq2 analysis. Significantly enriched bacterial ASVs classified at the genus level with P < 0.05 are listed. All P-values have been corrected for a false discovery rate. (**E**) Bacterial genera exhibiting the greatest differences in representation are depicted (log2 Fold Change).

We evaluated the main characteristics of the gut microbiota community in Auto-Tr and YT-tr groups at 18 months old (both groups subjected to FMT in middle age). We measured alpha-diversity, by observed richness and Shannon index. We observed a diversity increment in animals who received FMT from young and trained donors, nevertheless it did not reach statistical significance (Fig. 7A). On the other hand, beta diversity evaluated by Unweighted UniFrac showed significant differences between the microbial communities (permANOVA p=0.011) (Fig. 7B). Bray-Curtis dissimilarity showed similar results (Fig. S6A). A comprehensive examination of the gut microbiota (Fig. 7C-7D) revealed a notable enrichment of various amplicon sequence variants (ASVs) associated with the genera *Prevotellaceae_UCG-001* and *Alistipes*, among others, in YT-Tr aged mice. Employing a DESeq2 modelling and analysis, we additionally identified that the genera *Akkermansia* and *Peptococcaceae* exhibit significantly higher abundance in YT-Tr mice, while *Quinella* and *Lachnospiraceae* demonstrated elevated abundance in Auto-Tr mice (Fig. 7E). The observed increases in *Prevotellaceae_UCG-001* and *Akkermansia* in YT-Tr animals are consistent with previous evidence indicating that these bacterial taxa are associated with enhanced cognitive function during aging. *Prevotellaceae_UCG-001* has been linked to improved cognitive performance in aging individuals, with studies suggesting its potential role in promoting neuroprotection and cognitive resilience [33]. Additionally, *Akkermansia muciniphila*, known for its beneficial effects on gut barrier function and metabolic health, has also been implicated in cognitive enhancement, with higher abundance correlating with better cognitive outcomes and reduced risk of cognitive decline [34]. These findings underscore the potential cognitive benefits conferred by modulations in gut microbiota composition induced by fecal microbiota transplantation from young-trained donors.

Finally, utilizing the KEGG database, we assessed the predicted functional potential of the microbiota associated with YT-Tr in comparison to the Auto-Tr group. Among the pathways showing significant differences between the YT-Tr and Auto-Tr groups, steroid biosynthesis and stilbenoid, diarylheptanoid, and gingerol biosynthesis stand out (Supplementary Fig. 6B).

## DISCUSSION

FMT from young-trained donors could be a promising therapeutic approach for addressing age-related cognitive alterations. Our study provides robust evidence that FMT from young-trained donors enhances cognitive function and synaptic plasticity in aged mice, reduces gut permeability and increases colonic ZO-1 levels, decreases inflammation, elevates fecal SCFA levels, and modifies the composition of the gut microbiota in a manner associated with improved cognitive function.

The cognitive function of older animals improved after receiving FMT from young-trained donors, which agrees with previous findings that relate the influence of gut microbiota on cognitive function. For example, individuals with specific microbial communities in their gut, including genera *Odoribacter, Butyricimonas*, and *Barnesiella*, tend to exhibit improved cognitive performance, particularly in memory and attention-related domains [35, 36].

Moreover, specific gut bacteria have been identified as producers of neurotransmitters like serotonin, dopamine, and gamma-aminobutyric acid (GABA) that are crucial for regulating mood, cognition, and behavior [37]. Dysbiosis has been linked to increased inflammation that correlates with cognitive decline and neurodegenerative diseases among older adults [2]. The bidirectional communication between the gut and brain, known as the gut-brain axis, provides a mechanistic framework for understanding how signals from the gut microbiota can modulate brain function and behavior [5]. Our current study shows that an FMT protocol from young-trained donors to aged animals can modulate the gut-brain axis. This is evidenced by the significant improvements in recognition memory, assessed through the NOR test, and spatial memory, evaluated with the OLM test, compared to old animals who received FMT from age-matched controls. We studied the role of synaptic plasticity through long-term potentiation (LTP), which is proposed as the potential mechanism underlying processes as memory and learning [18]. We observed an enhanced LTP at CA3-CA1 synapses in aged mice receiving FMT from young-trained donors, suggesting that changes in the gut microbiota composition during aging may impair hippocampal function. We also identified that synaptic regulators, such as PSD95 and FNDC5/Irisin [23, 24, 38] were upregulated in the hippocampus of old mice that received FMT from young-trained donors. These findings support previous research linking gut microbiota modulation to alterations in synaptic plasticity and cognitive function [39]. In addition, neonatal microbial colonization with *Bifidobacterium* caused normal synaptic density and neuronal activity, with a reduction in markers of reactive microglia, in contrast to the increased synaptic density, decreased firing rate, and microglia reactivity observed in the hippocampus and cerebellum of germ-free mice [40]. Recent evidence reports that antibiotics-induced dysbiosis in newborn mice leads to behavioral impairment by decreasing adult neurogenesis and hippocampal LTP and altered gene expression profile in the hippocampus [41]. Our findings suggest that the gut microbiota may have therapeutic potential by addressing cognitive alterations associated with aging-related hippocampal dysfunction.

The modulation of gut microbiota to improve cognitive health is increasingly recognized as crucial in the management of neurodegenerative diseases, including AD and Parkinson disease [42, 43]. Several studies have shown notable changes in the intestinal microbiome of individuals with dementia. These changes include reduced microbial diversity, dysbiosis characterized by imbalance in specific bacterial taxa including Firmicutes/Bacteroidetes ratio, and increased levels of inflammation associated microbes like certain species of Proteobacteria [42, 43]. AD patients exhibited a positive correlation between pro-inflammatory blood cytokines and the presence of *Escherichia/Shigella*, while a negative correlation was found with *Eubacterium rectale* [44]. These microbiome shifts often match up with cognitive decline and/or disease progression. Interventions aiming to reshape the gut microbiota have shown promising results in enhancing cognitive impairments in neurodegenerative conditions. For instance, clinical trials using 12-week probiotic supplementation with *Bifidobacterium longum*, *Lactobacillus rhamnosus*, and *Bifidobacterium breve* A1, have shown improved cognitive function in patients with AD or cognitive decline [45, 46].

FMT from healthy donors has emerged as a potential therapy for dementia or cognitive deterioration. Case reports and small studies have reported cognitive improvements following FMT [47, 48]. Advancements in FMT techniques, including capsules containing lyophilized microbiota, have facilitated its implementation [13]. This approach provides a practical and non-invasive way to administrate microbial therapy, making FTM more accessible and feasible as a treatment option.

Our study also revealed significant alterations in the gut microbiota composition of aged mice receiving FMT from young-trained donors. Specifically, enrichment of bacterial taxa associated with beneficial effects over cognitive function, such as *Prevotellaceae_UCG-001*, *Odoribacter*, and *Akkermansia*, were observed in aged mice subjected to FMT from young-trained donors. The observed increment of *Prevotellaceae_UCG-001* and *Akkermansia* in aged mice receiving FMT from young-trained donors is interesting, because both bacterial taxa have been implicated in cognitive resilience and neuroprotection promotion. *Prevotellaceae_UCG-001* shown to improve cognitive performance in aging individuals [33], while *Akkermansia* has been linked to enhanced gut barrier function, reduced inflammation, and improved cognitive outcomes [34]. Additionally, supplementation with *Akkermansia muciniphila* has prevent synaptic reduction and microglia activation in the hippocampus of a sleep deprivation murine model [49]. It is important to note that *Akkermansia* is more present in young individuals (humans and mice) and is related to healthy aging [50, 51]. *Akkermansia* was also shown to have increased abundance in the gut microbiota of our young-trained donors. *Odoribacter*, other taxa overrepresented in YT-tr group, has been inversely associated with cognitive impairment and was positively associated with hippocampal volume in humans [35]. We also found that *Quinella* was one of the most overrepresented genera in the Auto-Tr group, this class of bacteria has been linked to pathological conditions like AD [52]. The KEGG analysis infers that steroid, stilbenoid, diarylheptanoid, and gingerol biosynthesis increased in YT-Tr mice. The steroid biosynthesis pathway includes the synthesis of molecules such as cholecalciferol, ergocalciferol, and phytosterol, all of these compounds have been associated with improved cognition in older individuals [53, 54]. Supplementation with stilbenoids (such as resveratrol), diarylheptanoids (such as curcumin), and gingerol has also been related to positive cognitive outcomes [55]. In future research, it would be important to study the role of these pathways and molecules in a therapeutic FMT context. Taken together, our data underscore the potential cognitive benefits conferred by modulation of the gut microbiota composition induced by FMT from young-trained donors.

Furthermore, the increments in cecal levels of SCFAs after FMT from young-trained donors provide additional insight into the mechanisms that underlie the cognitive benefits of gut microbiota modulation in old mice. SCFAs, including butyrate, propionate, and valerate, support the gut barrier integrity, reduce inflammation, and promote cognitive function [5]. Butyrate treatment was able to promote LTP and depotentiation and increase the abundance of dendritic spines in AD mice [56]. Furthermore, synapse-associated proteins (as PSD-95, SYP, NR2B) were increased and the pro-inflammatory cytokines (TNF-α, IL-6, IL-1β) were reduced in the AD animals treated with butyrate [56]. Among the bacteria genera we found overrepresented in YT-tr group and YT donors, *Akkermansia* and *Alistipes* are proposed as propionate-producing bacteria [57, 58], *Odoribacter* (as *Odoribacter splanchnicus*) produces butyrate and propionate [59], and *Prevotellaceae_UCG-001* is positively associated to propionate and valerate levels in human fecal samples [60]. The observed increase in SCFA levels in the cecal content of aged mice receiving FMT from young-trained donors suggests that alterations in gut microbiota and SCFA production may contribute to the cognitive enhancements observed in this group.

In addition to cognitive improvements, FMT from young-trained donors was associated with a significant reduction in gut permeability, higher levels of colonic ZO-1, and an improvement in the inflammatory state in aged mice. This reduction in gut permeability has been linked to improvements in cognitive function. An intervention with 40 participants found that increased gut permeability (estimated by elevated LPS serum levels) was associated with a decline in global cognitive performance [61]. Another research observed a negative correlation between the severity of the gut barrier dysfunction and cognitive function, evaluating patients with AD, mild cognitive impairment, and normal control cases [62]. In aged mice, increased gut permeability induced by sevoflurane anesthesia leads to a raised in serum proinflammatory cytokines, activation of microglia, and TLR4/NF-κB signaling pathway in the prefrontal cortex, with cognitive impairment and emotional phenotype abnormality [63]. The reduction in intestinal permeability could be directly related to a decrease in inflammation levels, as evidenced by our results, in which we found reduced gut permeability and diminished serum levels of proinflammatory cytokines in aged mice receiving FMT from young-trained donors. Compared to controls, animals that received FMT from young-trained donors showed lower serum levels of different proinflammatory molecules (IL-1α, IL-1β, IL-3, IL-6, RANTES, TNF-α, MIP-1α, and IL-17A) that have been associated to reduced cognitive performance [64], meanwhile GM-CSF, IL-2, and IL-10 serum levels were elevated in YT-Tr mice compare to Auto-Tr. Those factors have been associated with positive cognitive function [29, 30, 65]. Some studies have shown that interventions targeting the gut microbiota, such as prebiotic or probiotic supplementation, can improve gut barrier function and reduce systemic inflammation, enhancing cognitive performance in older subjects. For instance, in a murine AD model the use of prebiotic fructo-oligosaccharides for 4 weeks improved learning and memory functions, restored gut microbiota diversity, maintained healthy cell morphology in the small intestine, reduced cytokine levels and increased secretion of neuroprotective neurotransmitters [66]. A randomized placebo-controlled trial with patients older than 60 years old, the administration of a probiotic capsule (*Bifidobacterium longum*, *Lactobacillus acidophilus*, and *Enterococcus faecalis*) during hospitalization, prevented the incidence of postoperative cognitive impairment and decreased the levels of plasma IL-6 and cortisol compared to placebo group [67]. These findings and our results underscore the therapeutic implications of targeting gut microbiota to improve gut permeability and reduce inflammation to promote healthy aging and preserve cognitive function. Although translating these findings to human treatments indeed holds significant potential, it also presents several challenges and considerations. The use of FMT from young, physically active donors could be explored as a therapeutic strategy to mitigate age-related cognitive decline in humans. However, this would require carefully designed clinical trials to assess safety, efficacy, and optimal protocols for donor selection and FMT administration. Ethical considerations include ensuring donor and recipient safety, informed consent, and addressing potential risks associated with transferring microbiota between individuals, such as the transmission of infectious agents or unintended metabolic effects. Regulatory hurdles must also be navigated, as FMT is currently approved primarily for treating recurrent *Clostridium difficile* infections [13]. Additionally, differences between human and murine physiology necessitate cautious interpretation of preclinical results. Future research should focus on identifying specific microbial taxa or metabolites responsible for the cognitive benefits observed, which could lead to targeted probiotic or dietary interventions with fewer ethical and regulatory barriers. Collaborations between researchers, clinicians, and regulatory bodies will be essential to advance from basic research to clinical applications, ensuring that scientific rigor and ethical standards are upheld throughout the translational process.

One limitation of our study is we did not include young and aged mice groups without any intervention. We selected young and trained mice as fecal donors to maximize the positive effects of young adulthood and exercise on gut microbiota. We aimed to investigate the effects of FMT from these donors on aged animals, comparing it to an appropriate control group in which aged animals received FMT from other aged animals. Although we included a group of aged animals (18 months old) that received FMT from sedentary young donors (YS-Tr) to isolate the effect of exercise, FMT from young-trained donors resulted in superior outcomes in behavioral tasks, hippocampal electrophysiological evaluations, and elevated levels of hippocampal synaptic regulators associated with improved cognition compared to the YS-Tr group. Additionally, this study only involved male donors and recipients. Therefore, it would be valuable for future studies to evaluate whether similar results are observed in females receiving FMT from female donors or if the donor's sex significantly influences the effects of FMT on the cognitive function of the recipient. Another limitation of our study is that we did not assess whether the cognitive improvements observed are sustained over long periods. Our FMT protocol involved administering FMT to 12-month-old animals, followed by reinforcement at 15 months of age, with behavioral and physiological assessments conducted around 18 months of age. Additionally, we performed FMT on 18-month-old mice, with behavioral and electrophysiological assessments conducted at 20 months of age. In both cases, analyses were performed 1-2 months after the FMT regimen was completed. Longitudinal studies tracking the mice over extended periods would provide valuable insights into the durability of the treatment effects. Future research should aim to evaluate the long-term efficacy and stability of the cognitive improvements induced by FMT, as well as the persistence of gut microbiota alterations and their correlation with sustained cognitive benefits.

Our work highlights the therapeutic potential of FMT from young-trained donors in mitigating age-related cognitive decline and confirms the intricate crosstalk in the gut-brain axis, where the gut microbiota, intestinal barrier function, inflammation, and cognitive health are the main elements. Future research should further elucidate the underlying mechanisms within the brain and therapeutic implications of FMT in cognitive aging, with the goal of developing novel therapeutic interventions to promote healthy cognitive aging and improve the quality of life for older adults.

## Supplementary Materials

The Supplementary data can be found online at: www.aginganddisease.org/EN/10.14336/AD.2024.1089.

## Data Availability

The authors declare that all data are available from the corresponding authors upon request.
