# Supplementary material for: Fecal Microbiota Transplantation from Young-Trained Donors Improves Cognitive Function in Old Mice Through Modulation of the Gut-Brain Axis
Source: Aging Dis. 2024 Dec 26;16(6):3649–70. doi: 10.14336/AD.2024.1089 (PMC12539541; doi:10.14336/AD.2024.1089)
Supplement: Supplementary file 1 — The Supplementary data can be found online at: www.aginganddisease.org/EN/10.14336/AD.2024.1089. [file AD-16-6-3649-s.pdf]

SUPPLEMENTARY DATA

Fecal Microbiota Transplantation from Young-Trained Donors Improves Cognitive Function in Old Mice Through Modulation of the Gut-Brain Axis

Camila Cerna, Nicole Vidal-Herrera, Francisco Silva-Olivares, Daniela Álvarez, Camila González-Arancibia, Miltha Hidalgo, Pabla Aguirre, José González-Urra, Camila Astudillo-Guerrero, Michel Jara, Omar Porras, Gonzalo Cruz, Christian Hodar, Paola Llanos, Pamela Urrutia, Claudia Ibacache-Quiroga, Yulia Nevzorova, Francisco Javier Cubero, Marco Fuenzalida, Samanta Thomas-Valdés, Gonzalo Jorquera

© 2024. Serna C et al. Published online at http://www.aginganddisease.org/EN/10.14336/AD.2024.1089

SUPPLEMENTARY DATA

Supplementary Table 1. Primer sequences and thermocycling conditions for the qPCR experiments.

Gene	Primers	Thermocycling conditions
Bdnf	Forward 5'-TGGCCCTGCGGAGGCTAAGT-3'<br>Reverse 5'-AGGGTGCTTCCGAGCCTTCCT-3'	95°C for 10 minutes, followed by 40 cycles of: 95°C for 10 seconds, 60°C for 15 seconds, and 72°C for 15 seconds
Emr1	Forward 5'-AATCGCTGCTGGTTGAATACAG-3'<br>Reverse 5'-CCAGGCAAGGAGGACAGAGTT-3'	95°C for 10 minutes, followed by 40 cycles of: 95°C for 10 seconds, 54°C for 15 seconds, and 72°C for 15 seconds
Gfap	Forward 5'-AACGACTATCGCCGCCAACTG-3'<br>Reverse 5'-CTCTTCCTGTTCGCGCATTTG-3'	95°C for 10 minutes, followed by 40 cycles of: 95°C for 10 seconds, 55°C for 60 seconds, and 72°C for 30 seconds
Il-10	Forward 5'-GCTCTTACTGACTGGCATGAG-3'<br>Reverse 5'-CGCAGCTCTAGGAGCATGTG-3'	95°C for 10 minutes, followed by 40 cycles of: 95°C for 10 seconds, 60°C for 15 seconds, and 72°C for 15 seconds
Tnf-α	Forward 5'-ACGGCATGGATCTCAAAGAC-3'<br>Reverse 5'-AGATAGCAAATCGGCTGACG-3'	95°C for 10 minutes, followed by 40 cycles of: 95°C for 10 seconds, 60°C for 15 seconds, and 72°C for 15 seconds
Il-1β	Forward 5'-GCAACTGTTCCTGAACTCAACT-3'<br>Reverse 5'-ATCTTTTGGGGTCCGTCAACT-3'	95°C for 10 minutes, followed by 40 cycles of: 95°C for 10 seconds, 60°C for 15 seconds, and 72°C for 15 seconds
Rplp0	Forward 5'-CTCCAAGCAGATGCAGCAGA-3'<br>Reverse 5'-ATAGCCTTGCGCATCATGGT-3'	95°C for 10 minutes, followed by 40 cycles of: 95°C for 10 seconds, 60°C for 15 seconds, and 72°C for 15 seconds

© 2024. Serna C et al. Published online at http://www.aginganddisease.org/EN/10.14336/AD.2024.1089

SUPPLEMENTARY DATA

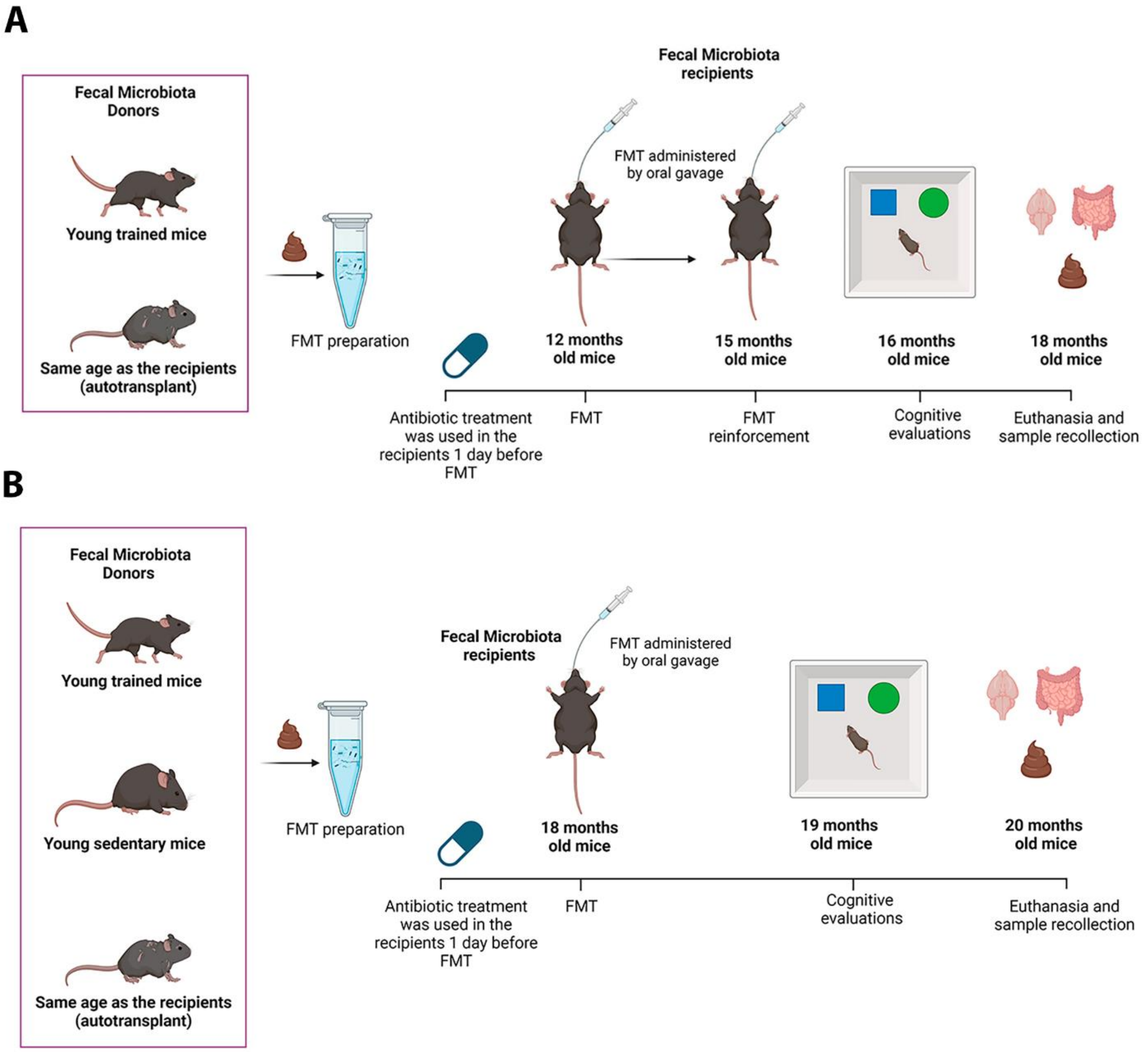

Supplementary Figure 1. Experimental design. (A) Twelve-month-old male mice were subjected to antibiotic treatment (ampicillin at 1 mg/mL, vancomycin at 5 mg/mL, neomycin trisulfate at 10 mg/mL, and metronidazole at 10 mg/mL) via oral gavage. The following day, they received fecal microbiota transplantation (FMT) for three consecutive days during the first week, followed by once-weekly administrations for three weeks. Finally, at 15 months of age, FMT was reinforced with three additional doses administered over three consecutive days. FMT donors were either young and trained male mice or the same age as the recipient mice. Behavioral tests were conducted when mice reached 16 months of age, followed by euthanasia and tissue collection at 18 months of age. (B) Eighteen-month-old male mice were subjected to antibiotic treatment (same as described above) via oral gavage. The following day, they received fecal microbiota transplantation (FMT) for three consecutive days during the first week, followed by once-weekly administrations for three weeks. FMT donors were either young and trained male mice, young and sedentary male mice, or the same age as the recipient mice. Behavioral tests were conducted at 19 months of age, followed by euthanasia and tissue collection at 20 months of age.

© 2024. Serna C et al. Published online at http://www.aginganddisease.org/EN/10.14336/AD.2024.1089

SUPPLEMENTARY DATA

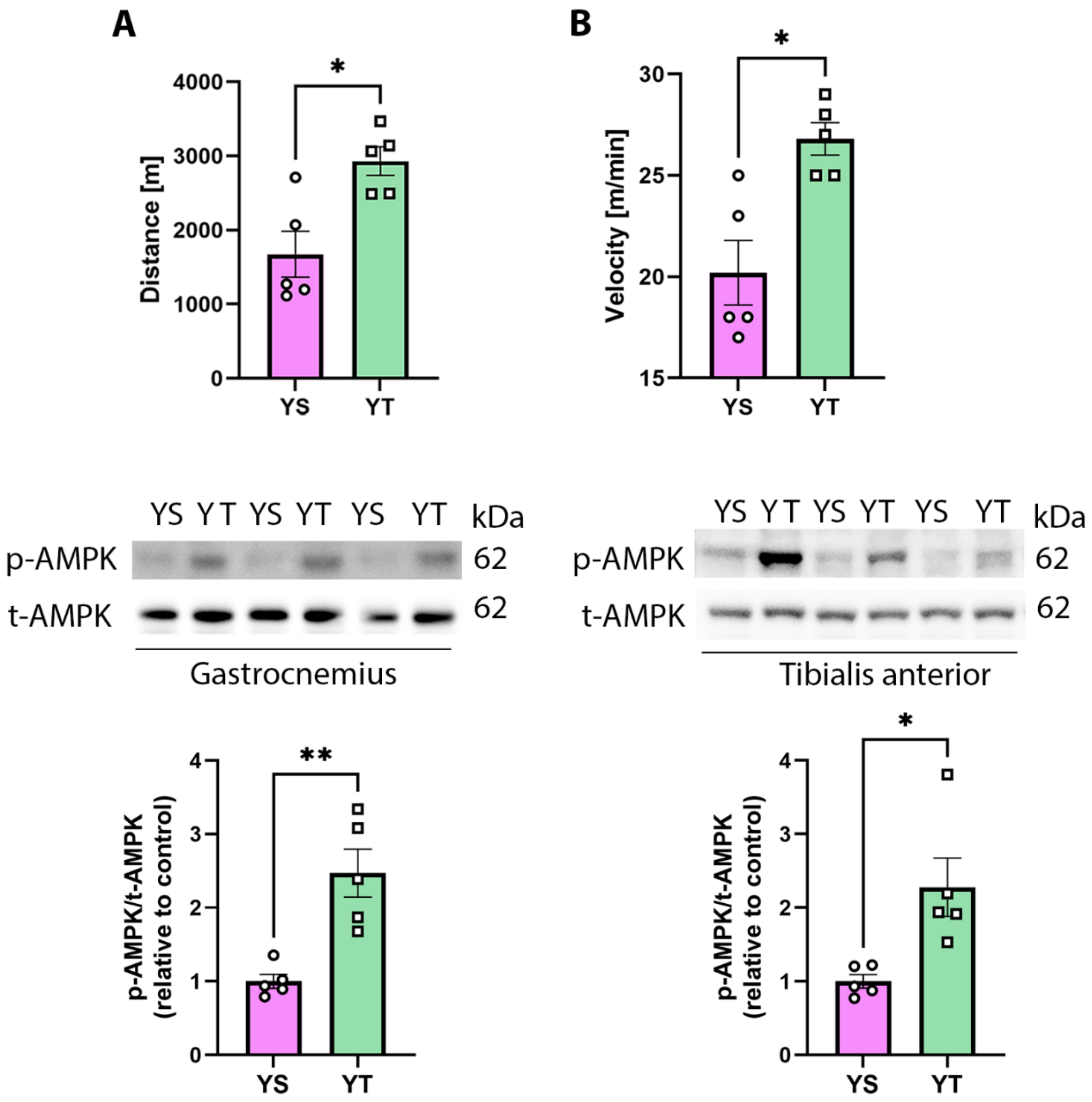

Supplementary Figure 2. Young mice experience biological adaptations to a chronic exercise routine. Three-month-old male mice were trained on a treadmill for six weeks, one hour per day, five days per week, at 60% of their maximum running speed. After this period, they exhibited increased running distance (A) and velocity (B) during a fatigue test compared to sedentary animals (not subjected to training). (C) In the gastrocnemius and tibialis anterior muscles, trained animals displayed increased AMPK phosphorylation (Thr172) compared to sedentary ones. YS: Young sedentary, YT: Young trained. Data are presented as means ± SEM; N=5 for the YS group and N=5 for the YT group. *p < 0.05 and **p < 0.01, Mann-Whitney test.

SUPPLEMENTARY DATA

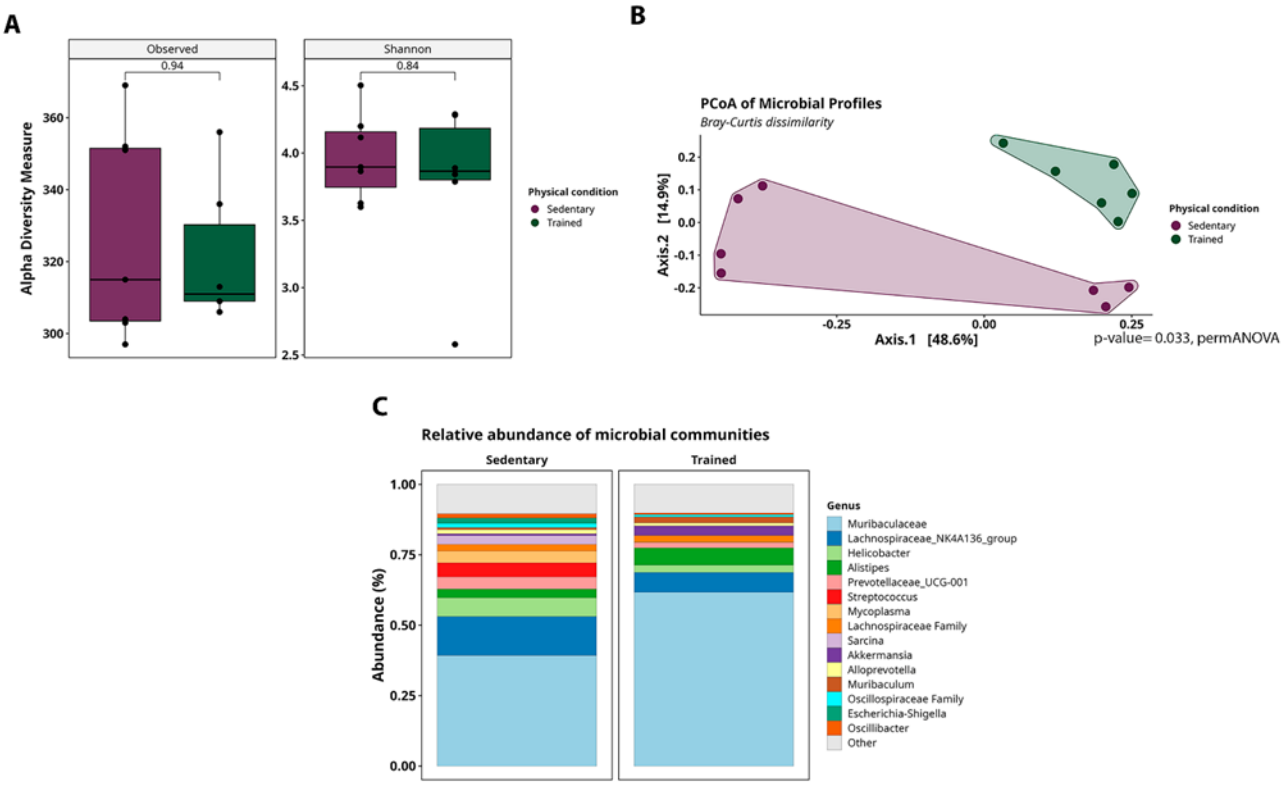

Supplementary Figure 3. The gut microbiome of young, trained mice exhibits a different composition compared to that of young, sedentary mice. (A) On the left, the observed count of enriched ASVs is compared between trained (N = 6) and sedentary (N = 7) groups, with data presented as the precise number of ASVs. On the right, the Shannon index is assessed for both trained (N = 6) and sedentary (N = 7) groups, showing no significant differences between them. (B) PCoA of beta diversity, measured using Bray-Curtis dissimilarity, demonstrates distinct bacterial community compositions between trained (N = 6) and sedentary (N = 7) groups (permANOVA, p = 0.033). (C) The mean relative abundance of genera is compared between groups, with genera contributing less than 1% relative abundance grouped together and labeled as "others."

© 2024. Serna C et al. Published online at http://www.aginganddisease.org/EN/10.14336/AD.2024.1089

SUPPLEMENTARY DATA

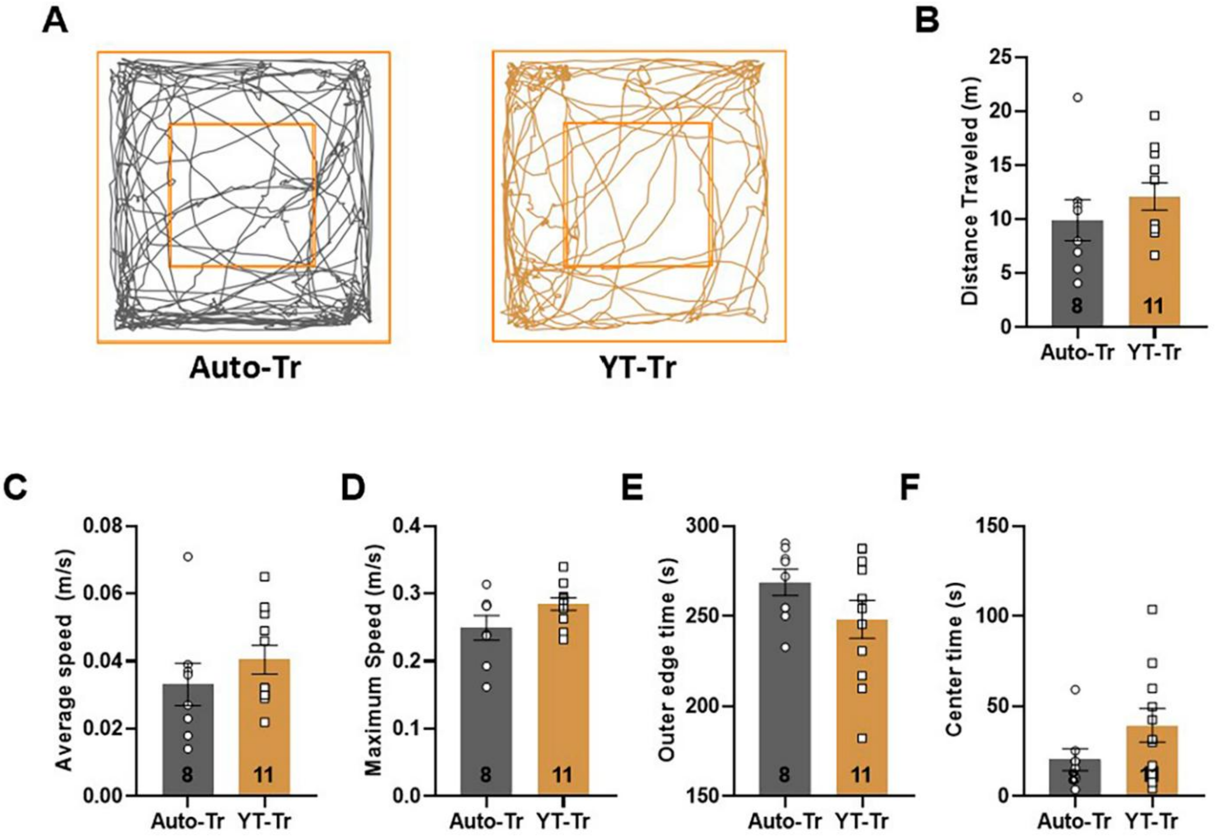

Supplementary Figure 4. FMT did not alter motor activity or anxious-like behaviors in old mice. (A) Tracking plot of a representative animal of each group during the open field test. Locomotor parameters, including distance traveled (B), average speed (C), and maximum speed (D), were measured and presented in bar graphs. The time spent by mice in the outer and center edges was also recorded and shown in (E) and (F), respectively. No differences were observed between the groups. Data are presented as means ± SEM, N=8 for Auto-Tr group and N=11 for YT-Tr. Data collected from individual mice were analyzed using the Mann Whitney test.

© 2024. Serna C et al. Published online at http://www.aginganddisease.org/EN/10.14336/AD.2024.1089

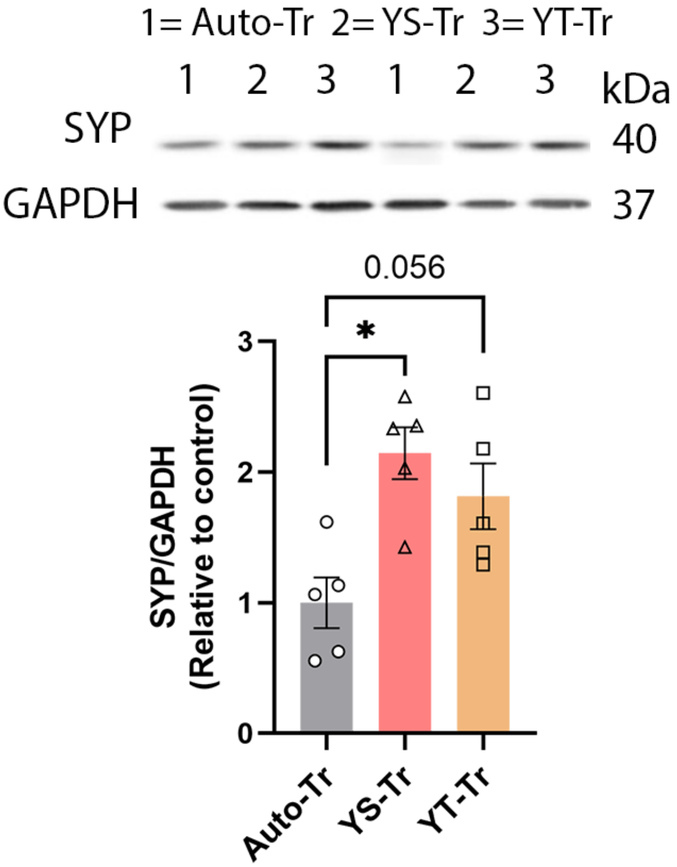

Supplementary Figure 5. SYP levels were increased in the hippocampal tissue of YS-Tr and YT-Tr mice compared to the Auto-Tr group. Protein levels of synaptic plasticity modulator Synaptophysin (SYP) was measured by immunoblot in the hippocampus of Auto-Tr, YS-Tr, and YT-Tr mice (N = 5) that received FMT at 18 months of age. GAPDH (37 kDa) was used as a control for total protein content. Data are presented as means ± SEM; *$p < 0.05$, as determined by the Kruskal–Wallis test.

© 2024. Serna C et al. Published online at http://www.aginganddisease.org/EN/10.14336/AD.2024.1089

SUPPLEMENTARY DATA

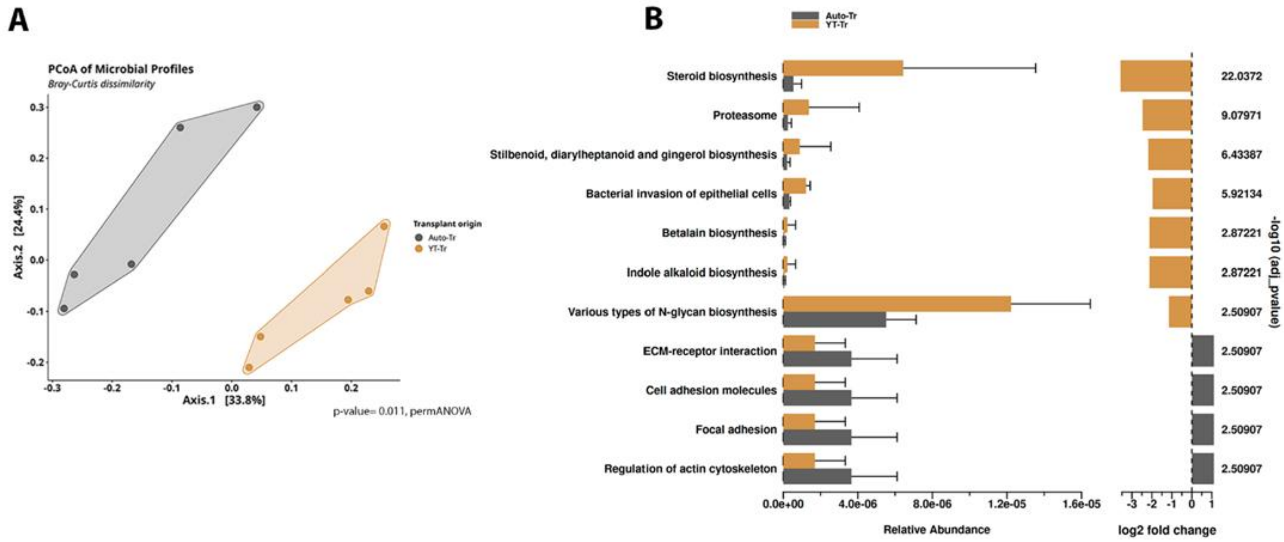

Supplementary Figure 6. Beta diversity and KEGG pathway enrichment analysis. (A) PCoA of beta-diversity of Auto-Tr (N = 5) and YT-Tr (N = 5) as measured by Bray-Curtis dissimilarity. The bacterial communities in Auto-Tr and YT-Tr are dissimilar to each other, permANOVA p=0.011. (B) KEGG pathway prediction of metagenome functional content from 16S rRNA. Relative abundance and fold change for each predicted functional category in KEGG pathways between Auto-Tr and YT-Tr groups was calculated by edgeR differential abundance analysis, using ggpicrus2 R package, with significance threshold of $p < 0.05$.

© 2024. Serna C et al. Published online at http://www.aginganddisease.org/EN/10.14336/AD.2024.1089

SUPPLEMENTARY DATA

© 2024. Serna C et al. Published online at http://www.aginganddisease.org/EN/10.14336/AD.2024.1089
